# Sesquilignans and sesquiterpenoid from the stem barks of *Illicium simonsii* and their anti-AChE activity

**DOI:** 10.1007/s13659-012-0026-z

**Published:** 2012-05-24

**Authors:** Chuan-Fu Dong, Lei Liu, Huai-Rong Luo, Xiao-Nian Li, Zheng-Ye Guan, Yi-Fen Wang

**Affiliations:** 1 State Key Laboratory of Phytochemistry and Plant Resources in West China, Kunming Institute of Botany, Chinese Academy of Sciences, Kunming, 650201 China; 2 Graduate University of Chinese Academy of Sciences, Beijing, 100049 China

**Keywords:** *Illicium simonsii*, sesquilignans, sesquiterpenoid, anti-AChE activity

## Abstract

Three new sesquilignans, **1–3**, a new sesquiterpenoid, **4**, and three known compounds were isolated from the stem barks of *Illicium simonsii*. The structures of new compounds (**1–4**) were elucidated by spectroscopic methods. A biosynthetic pathway was proposed for simonsienols A-C (**1–3**). Anti-AChE activity and anti-BuChE activity were evaluated for all compounds except for *α*-cadinol ethyl ether (**4**). As a result, isodunnianol (**7**) exhibited anti-AChE activity with an IC_50_ value of 13.0 µM.

**Electronic Supplementary Material:**

Supplementary material is available for this article at 10.1007/s13659-012-0026-z and is accessible for authorized users.

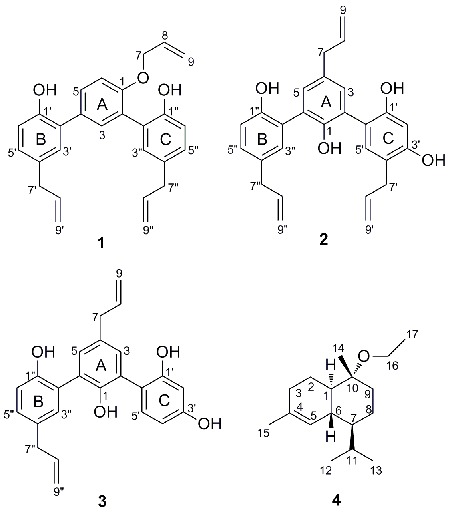

## Introduction

The genus *Illicium* is the only member of the family Illiciaceae and is an evergreen shrub or tree. About 40 species have been found disjunctively in eastern North America, Mexico, the West Indies and the eastern Asia. The highest concentration of species is in the northern Myanmar and the southern China where nearly 35 species have been described.[1,2] *seco*-Prezizaane-type sesquiterpenes, prenylated $C_6$-$C_3$ compounds and sesqui-neolignans are the secondary metabolites characteristic of the *Illicium* plants.[3] 4-epi-illicinone *E*-12-shikimate and 3-hydroxyillifunone B were isolated from the fruits of *I. simonsii*.[4] Simonin A and 1-hydroxyl-2-*O*-$\beta$-D-6′-acetyl-glucopyranosyl-4-allybenzene were isolated from the stems of *I. simonsii* and simonin A showed activity against oral microbial organisms.[5] The unique structures and interesting biological activities have lured us to extensively investigate on the bioactive compounds of *I.* species. As a result, three new sesquilignans (**1**–**3**) and one new sesquiterpenoid (**4**), together with three known compounds macranthol (**5**),[6] dunnianol (**6**),[6] and isodunnianol (**7**)[7] were isolated from the stem barks of *I. simonsii.* Herein we report the isolation, structure elucidation and anti-AChE activity of these compounds.

## Results and Discussion

The aired-dried stem barks of *I. simonsii* were extracted with EtOH for three times at room temperature. The extract was partitioned successively with petroleum ether, $CHCl_3$ and *n*-BuOH. The $CHCl_3$ fraction was subjected to a multistep chromatographic separation and purification procedures to afford pure compounds **1**–**7**.

The molecular formula of **1** was assigned as $C_{27}H_{26}O_3$ on the basis of HRESIMS at m/z 399.1965 $[M + H]^+$ (calcd. for $C_{27}H_{27}O_3$, 399.1960), indicating 15 degrees of unsaturation. Its IR spectrum showed the presence of hydroxyl group (3418 $cm^{-1}$), aromatic ring (1638, 1495, 1424 $cm^{-1}$) and ether linkage (1281, 1229 $cm^{-1}$). The $^1H$ NMR spectra (Table 1) of **1** showed signals due to three allyl groups [$\delta_H$ 4.68 (2H, d, $J$ = 5.3 Hz, H-7), 6.01 (1H, ddd, $J$ = 5.3, 10.5, 17.3 Hz, H-8), 5.38 (1H, dd, $J$ = 1.7, 17.3 Hz, H-9a), 5.30 (1H, dd, $J$ = 1.5, 10.5 Hz, H-9b); 3.37 (2H, d, $J$ = 6.6 Hz, H-7′), 5.97 (1H, ddd, $J$ = 6.6, 8.4, 18.0 Hz, H-8′), 5.10 (1H, dd, $J$ = 1.8, 18.0 Hz, H-9′a), 5.07 (1H, dd, $J$ = 1.8, 8.4 Hz, H-9′b); 3.35 (2H, d, $J$ = 6.8 Hz, H-7″), 5.99 (1H, ddd, $J$ = 6.8, 8.4, 18.0 Hz, H-8″), 5.06 (1H,

*To whom correspondence should be addressed. E-mail: wangyifen@mail.kib.ac.cn

Springer

**Table 1.** $^1$**H NMR data of 1 ($CDCl_3$), 2 (acetone-$d_6$) and 3 (acetone-$d_6$)**

| | 1 | 2 | 3 |
|---|---|---|---|
| position | $\delta_H$ (*J* in Hz) | $\delta_H$ (*J* in Hz) | $\delta_H$ (*J* in Hz) |
| 3 | 7.49 (d, 2.3) | 7.12 (d, 2.6) | 7.14 (d, 2.6) |
| 5 | 7.48 (d, 2.3, 8.8) | 7.11 (d, 2.6) | 7.13 (d, 2.6) |
| 6 | 7.14 (d, 8.8) | | |
| 7 | 4.68 (d, 5.3) | 3.42 (d, 6.8) | 3.42 (d, 6.8) |
| 8 | 6.01 (ddd, 5.3, 10.5, 17.3) | 6.03 (ddd, 6.8, 10.5, 18.0) | 6.04 (ddd, 6.8, 16.8, 17.1) |
| 9a | 5.38 (dd, 1.7, 17.3) | 5.11 (dd, 1.9, 18.0) | 5.14 (dd, 2.1, 16.8) |
| 9b | 5.30 (dd, 1.5, 10.5) | 5.08 (dd, 1.9, 10.5) | 5.09 (dd, 1.8, 17.1) |
| 1′ | | | |
| 2′ | | 6.66 (s) | 6.81 (d, 3.0) |
| 3′ | 7.11 (d, 2.5) | | |
| 4′ | | | 6.75 (dd, 8.6, 3.0) |
| 5′ | 7.12 (d, 2.5, 8.8) | 6.67 (s) | 6.85 (d, 8.6) |
| 6′ | 6.99 (d, 8.8) | | |
| 7′ | 3.37 (d, 6.6) | 3.40 (d, 6.8) | |
| 8′ | 5.97 (ddd, 6.6, 8.4, 18.0) | 6.01 (ddd, 6.8, 10.8, 15.8) | |
| 9′a | 5.10 (dd, 1.8, 18.0) | 5.15 (dd, 1.9, 10.8) | |
| 9′b | 5.07 (dd, 1.8, 8.4) | 5.13 (dd, 1.9, 15.8) | |
| 3″ | 7.08 (d, 2.4) | 7.14 (d, 2.3) | 7.12 (d, 2.2) |
| 5″ | 7.07 (d, 2.4, 8.8) | 7.09 (dd, 2.3, 8.3) | 7.07 (dd, 8.2, 2.2) |
| 6″ | 6.91 (d, 8.8) | 6.95 (d, 8.3) | 6.92 (d, 8.2) |
| 7″ | 3.35 (d, 6.8) | 3.37 (d, 6.8) | 3.36 (d, 6.8) |
| 8″ | 5.99 (ddd, 6.8, 8.4, 18.0) | 5.98 (ddd, 6.8, 10.5, 18.0) | 5.99 (ddd, 6.8, 10.1, 10.2 ) |
| 9″a | 5.06 (dd, 1.8, 18.0) | 5.03 (dd, 1.9, 18.0) | 5.04 (dd, 2.2, 10.2) |
| 9″b | 5.05 (dd, 1.8, 8.4) | 5.00 (dd, 1.9, 10.5) | 5.01 (dd, 1.8, 10.1) |

dd, *J* = 1.8, 18.0 Hz, H-9″a), 5.05 (1H, dd, *J* = 1.8, 8.4 Hz, H-9″b)] and three 1,2,4-trisubstituted benzene rings [$\delta_H$ 7.49 (1H, d, *J* = 2.3 Hz, H-3), 7.48 (1H, dd, *J* = 2.3, 8.8 Hz, H-5), 7.14 (1H, d, *J* = 8.8 Hz, H-6); 7.11 (1H, d, *J* = 2.5 Hz, H-3′), 7.12 (1H, dd, *J* = 2.5, 8.8 Hz, H-5′), 6.99 (1H, d, *J* = 8.8 Hz, H-6′); 7.08 (1H, d, *J* = 2.4 Hz, H-3″), 7.07 (1H, dd, *J* = 2.4, 8.8 Hz, H-5″), 6.91 (1H, d, *J* = 8.8 Hz, H-6″)]. The $^{13}$C NMR spectrum (Table 2) of **1** showed the presence of 27 carbons, which were categorized into nine quaternary, twelve methine and six methylene. HMBC correlations (Figure 1) of H-3″/C-5″, H-5″/C-1″, 3″, H-6″/C-1″, 2″, 4″, 5″, H-7″/C-3″, 4″, 5″ and $^1$H-$^1$H COSY correlation between H-5″ and H-6″ indicated that the position of the hydroxy group and allyl group on ring C should be at C-1″ and C-4″, respectively. Furthermore, HMBC correlations of H-3″/C-2 and H-3/C-2″ showed that ring C was connected to C-2 of ring A. Ring B was similarly confirmed to be connected to C-4 of ring A, indicating by the analyses of $^1$H-$^1$H COSY and HMBC spectras. HMBC correlations of H-3, 5, 6/C-1 ($\delta_C$ 154.0) and $^1$H-$^1$H COSY correlation between H-5 and H-6 indicated that an oxygenated group might be at C-1 of ring A. According to the above NMR analysis, **1** was indicated to be a sesquilignan, which was similar to macranthol (**5**).[6] The significant difference was that an allyl unit was connected to C-1 through an oxygen atom, which was confirmed by HMBC correlations of H-7 ($\delta_H$ 4.68)/C-1 ($\delta_C$ 154.0), 8, 9 and $^1$H-$^1$H COSY correlations of H-8/H-7, 9 (Figure 1). At last, the structure of **1** was established as shown and named simonsienol A.

Compound **2** gave a molecular formula of $C_{27}H_{26}O_4$ by HRESIMS. Analysis of its $^1$H NMR (Table 1) and $^{13}$C NMR (Table 2) data indicated that **2** was very similar to simonsinol[8] except for one more hydroxyl group at C-1′ in **2**, causing a significant downfield chemical shift of C-1′ ($\delta_C$ 151.5). HMBC correlations (Figure 1) of H-2′/C-4′, 6′, H-5′/C-6′, H-7′/C-3′ ($\delta_C$ 145.6), 4′, 5′ indicated that allyl group and the hydroxy group were connected to C-4′ and C-1′ of ring C, repectively. Thus, the structure of **2** was established as shown and named simonsienol B.

The molecular formula of **3** was determined as $C_{24}H_{22}O_4$ by HRESIMS. Analysis of its NMR (Tables 1 and 2) and MS data showed that **3** was similar to **2** except for the absence of one allyl group. Comparing with **2**, a significant upfield chemical shift of C-4′ ($\delta_C$ 118.0) was observed in **3**. HMBC correlations (Figure 1) of H-7/C-3, 4, 5, 8, 9, H-7″/C-3″, 4″, 5″, 8″, 9″, together with $^1$H-$^1$H COSY correlation between H-4′ and H-5′ on ring C further supported the above assignment. Thus, the structure of **3** was established as shown and named simonsienol C. A possible mechanism for the formation of **1**, **2** and **3** is shown in Scheme 1.

**Figure 1.** Selected $^1$H-$^1$H COSY and HMBC correlations of **1**, **2** and **3**

Compound **4** was obtained as yellowish gum. The molecular formula of **4** was determined as $C_{17}H_{30}O$ by positive HRESIMS. Analysis of its $^1$H NMR data (Experimental Section) revealed an ethyl group [$\delta_H$ 3.42–3.26 (2H, m, H-16), 1.15 (3H, t, *J* = 7.0 Hz, H-17)]. The $^{13}$C NMR data (Experimental Section) showed that **4** was a sesquiterpenoid, which was very similar to *α*-cadinol methyl ether[9] except for the presence of an ethyl group. Diagnostic HMBC correlations were observed between C-10 ($\delta_C$ 76.0), 17 and H-16, together with $^1$H-$^1$H COSY correlatons (Figure 2) of H-16/H-17, implying the connection through oxygen bridge between C-16 and C-10. **4** might be an artificial product which was produced through the reaction with EtOH during the extraction process. All the groups had the same orientations as those in *α*-cadinol

**Table 2.** $^{13}$**C NMR data of 1 ($CDCl_3$), 2 (acetone-$d_6$) and 3 (acetone-$d_6$)**

| position | 1 | 2 | 3 |
|---|---|---|---|
| | $\delta_C$ mult. | $\delta_C$ mult. | $\delta_C$ mult. |
| 1 | 154.0 (s) | 149.7 (s) | 149.9 (s) |
| 2 | 128.7 (s) | 128.2 (s) | 128.3 (s) |
| 3 | 129.6 (d) | 132.0 (d) | 131.9 (d) |
| 4 | 131.3 (s) | 133.4 (s) | 133.2 (s) |
| 5 | 133.3 (d) | 131.9 (d) | 131.7 (d) |
| 6 | 113.8 (d) | 128.4 (s) | 128.3 (s) |
| 7 | 70.2 (t) | 40.0 (t) | 40.0 (t) |
| 8 | 132.0 (d) | 138.9 (d) | 138.9 (d) |
| 9 | 118.6 (t) | 115.6 (t) | 115.7 (t) |
| 1′ | 152.0 (s) | 151.5 (s) | 151.8 (s) |
| 2′ | 125.9 (s) | 116.2 (d) | 116.4 (d) |
| 3′ | 131.3 (d) | 145.6 (s) | 147.6 (s) |
| 4′ | 132.6 (s) | 130.1 (s) | 118.0 (d) |
| 5′ | 129.6 (d) | 116.9 (d) | 118.6 (d) |
| 6′ | 117.8 (d) | 128.0 (s) | 127.8 (s) |
| 7′ | 39.4 (t) | 35.4 (t) | |
| 8′ | 137.6 (d) | 138.1 (d) | |
| 9′ | 115.6 (t) | 115.8 (t) | |
| 1″ | 150.8 (s) | 153.0 (s) | 153.2 (s) |
| 2″ | 127.2 (s) | 126.3 (s) | 127.0 (s) |
| 3″ | 130.3 (d) | 132.7 (d) | 132.6 (d) |
| 4″ | 132.4 (s) | 132.7 (s) | 132.5 (s) |
| 5″ | 128.7 (d) | 129.9 (d) | 129.7 (d) |
| 6″ | 115.8 (d) | 116.9 (d) | 117.3 (d) |
| 7″ | 39.4 (t) | 39.9 (t) | 40.0 (t) |
| 8″ | 137.7 (d) | 139.0 (d) | 139.1 (d) |
| 9″ | 115.7 (t) | 115.5 (t) | 115.5 (t) |

methyl ether, which was apparently confirmed by ROESY correlations (Figure 2) of H-1/H-16 and H-6/H-11. At last, **4** was characterized as $\alpha$-cadinol ethyl ether.

**Figure 2.** Selected $^1$H-$^1$H COSY, HMBC and NOESY correlations of **4**

All compounds except for compound **4** were evaluated for acetylcholinesterase (AChE) inhibitory activity by using 96-well microplate reader (680 XR, USA). Tacrine was used as a positive control with an $IC_{50}$ value of 0.33 $\mu$M. As a result, compound **7** was found to exhibit anti-AChE activity with an $IC_{50}$ value of 13.0 $\mu$M. However, all the tested compounds were inactive when BuChE inhibitory activity and neurotrophic effect were assayed.

## Experimental Section

**General Experimental Procedures.** Optical rotations were recorded on a JASCO P-1020 polarimeter. IR and UV spectra were recorded on a Bruker Tensor 27 FT-IR spectrometer and a Shimadzu UV2401PC spectrometer, respectively. 1D and 2D NMR spectra were recorded on Bruker Avance III 600, or AM400 MHz spectrometers with TMS as internal standard at

**Scheme 1.** Proposed biosynthetic pathway for **1**, **2** and **3**

room temperature. HRESIMS were recorded on a API QSTAR Pulsar 1 spectrometer. Column chromatography (CC) was performed on silica gel (100–200 mesh, Qingdao Marine Chemical Ltd., Qingdao, China), Sephadex LH-20 (Amersham Biosciences, Sweden) and RP-18 gel (40 × 75 $\mu$m, Fuji Silysia Chemical Ltd., Japan). Analytical and semipreparative HPLC were performed on SHIMADZU LC-20AT system equiped with Extend-C18 column (4.6 × 150 mm) and YMC-Pack ODS-A column (10 × 150 mm), respectively.

**Plant Materal.** Stem barks of *I. simonsii* were collected in Dongchuan of Yunnan province, China, in June 2010 and were identified by Dr. Rong Li of Kunming Institute of Botany, Chinese Academy of Sciences. A sample was deposited in our laboratory. A voucher specimen of *I. simonsii* (LSF011-12) is deposited in State Key Laboratory of Phytochemistry and Plant Resources in west China, Kunming Institute of Botany, Chinese Academy of Sciences.

**Extraction and Isolation.** The air-dried stem barks of *I. simonsii* (28 kg) were powdered and extracted with 90% EtOH (3 × 25 L) at room temperature. The EtOH extract was evaporated *in vacuo* to yield a dark black residue, which was successively fractionated with petroleum ether, $CHCl_3$ and *n*-BuOH. A portion of the $CHCl_3$ extract (1378 g) was separated by silica gel column chromatography using $CHCl_3$/MeOH (20:1 to 2:1) as a gradient solvent system to afford fractions 1–20.

Fraction 4 was subjected to a MCI column, eluted with a gradient of MeOH/$H_2O$ (80:20 to 95:5), to give eight subfractons, A1–A8. Fraction A5 was subjected to rapeated silica gel CC (petroleum ether/$Me_2CO$, 20:1 to 5:1) to afford four subfactions (A51–A54). Fraction A54 was chromatographed over a Sephadex LH-20 with MeOH and further purified by semipreparative HPLC to yield compound **1** (9.0 mg, $t_R$ 33.0 min, $CH_3CN/H_2O$ 70:30). Fraction 8 was eluted on RP-18 column with MeOH/$H_2O$ (40:60 to 70:30) to give two fractions. The first fraction was purified by Sephadex LH-20

to give **3** (40.0 mg) and **2** (35.0 mg). Fraction 9 was gradiently eluted with petroleum ether/$Me_2CO$ (20:1 to $Me_2CO$) to give B–D. Fraction B (6.0 g) was subjected to a RP-18 column eluting with a gradient of MeOH/$H_2O$ (70:30 to 95:5) and purified with petroleum ether/EtOAc (15:1) to yield **6** (755 mg). Fraction 12 was subjected to repeated silica gel CC (petroleum ether/$Me_2CO$, 90:10 to 70:30) to yield fraction E–H. Fraction G (5.0 g) was fractionated with a gradient of petroleum ether/EtOAc (95:5 to 80:20) to yield fractions $G_1$–$G_4$. Fraction $G_2$ was separated on Sephadex LH-20 eluting with MeOH to yield **4** (112.0 mg). Fraction 15 was gradiently eluted with $CHCl_3$/MeOH (20:1 to 2:1) to yield fractions I–N. Fraction L was eluted with petroleum ether/$Me_2CO$ (10:1 to 2:1) to give cristal **5** (297 mg). Fraction 18 was subjected to repeated silica gel CC ($CHCl_3$/MeOH, 40:1 to 5:1) to yield fractions O–R. Fraction Q was further separated by semipreparative HPLC purification with aqueous $CH_3CN$ to yield **7** (22.0 mg, $t_R$ 25.0 min, $CH_3CN/H_2O$ 26:74).

**Simonsienol A (1):** colorless gum; $[\alpha]_D^{18}$ – 10.3 (*c* 0.001, $CHCl_3$); UV ($CHCl_3$) $\lambda_{max}$ (log $\varepsilon$) 241 (3.84), 293 (3.52) nm; IR $\nu_{max}$ (KBr) 3418, 1638, 1495, 1424, 1281, 1229, 993, 914, 820 $cm^{-1}$; $^1H$ (600 MHz) and $^{13}C$ NMR (100 MHz) data ($CDCl_3$), see Tables 1 and 2; HRESIMS (positive): *m/z* 399.1965 ($[M + H]^+$, $C_{27}H_{27}O_3$, calcd. 399.1960).

**Simonsienol B (2):** colorless, amorphous solid; $[\alpha]_D^{26}$ – 2.0 (*c* 0.004, MeOH); UV ($CHCl_3$) $\lambda_{max}$ (log $\varepsilon$) 217 (3.91), 303 (3.21) nm; IR $\nu_{max}$ (KBr) 3426, 3073, 2974, 1638, 1599, 1467, 1429, 1282, 1174, 1064, 993, 910, 818, 730, 660 $cm^{-1}$; $^1H$ (600 MHz) and $^{13}C$ NMR (100 MHz) data (acetone-$d_6$), see Tables 1 and 2; HRESIMS (positive): *m/z* 415.1912 ($[M + H]^+$, $C_{27}H_{27}O_4$, calcd. 415.1909).

**Simonsienol C (3):** colorless, amorphous solid; $[\alpha]_D^{26}$ – 2.9 (*c* 0.003, MeOH); UV (MeOH) $\lambda_{max}$ (log $\varepsilon$) 218 (3.95), 305 (3.26) nm; IR $\nu_{max}$ (KBr) 3381, 2074, 1638, 1487, 1457, 1422, 1346, 1202, 992, 909, 820, 791, 730, 660 $cm^{-1}$; $^1H$ (600 MHz) and $^{13}C$ NMR (100 MHz) data (acetone-$d_6$), see Tables 1 and 2; HRESIMS (positive): *m/z* 375.1605 ($[M + H]^+$, $C_{24}H_{23}O_4$, calcd. 375.1596).

***α*-cadinol ethyl ether (4):** yellow gum; $[\alpha]_D^{18}$ – 2.9 (*c* 0.004, $CHCl_3$); IR $\nu_{max}$ (KBr) 2959, 2930, 2894, 2871, 2830, 1452, 1386, 1366, 1155, 1139, 1105, 1073 $cm^{-1}$; $^1H$ NMR ($CDCl_3$, 600 MHz) $\delta$ 5.55 (1H, d, $J$ = 4.6 Hz, H-5), 3.42–3.26 (2H, m, H-16), 2.29–2.21 (1H, m, H-6), 2.06–1.90 (3H, m, H-3 and H-11), 1.74–1.61 (5H, overlapped, H-1, H-9$\alpha$, and H-15), 1.59–1.47 (2H, m, H-2), 1.35–1.20 (4H, m, H-7, H-8, and H-9$\beta$), 1.15 (3H, t, $J$ = 7.0 Hz, H-17), 1.09 (3H, s, H-14), 0.88 (3H, d, $J$ = 7.0 Hz, H-12), 0.82 (3H, d, $J$ = 6.9 Hz, H-13); $^{13}C$ NMR ($CDCl_3$, 150 MHz) $\delta$ 42.6 (d, C-1), 20.9 (t, C-2), 31.7 (t, C-3), 133.3 (s, C-4), 125.7 (d, C-5), 33.2 (d, C-6), 43.9 (d, C-7), 19.3 (t, C-8), 32.1 (t, C-9), 76.0 (s, C-10), 26.9 (d, C-11), 21.9 (q, C-12), 15.5 (q, C-13), 23.4 (q, C-14), 23.9 (q, C-15), 55.1 (t, C-16), 16.4 (q, C-17); HRESIMS (positive): *m/z* 273.2198 ($[M + Na]^+$, $C_{17}H_{30}ONa$, calcd. 273.2194).

**Anti-AChE assay.** 20 $\mu$L of 6.25 mM, DTNB, 20 $\mu$L of 6.25 mM ATCI, 40 $\mu$L of 0.04U/100 $\mu$L and sample were dissolved in phosphate buffer and added in increasing order to each well of 96 well plate and the absorbance was read at 405 nm.[10] Seven concentrations (1 $\mu$M, 3 $\mu$M, 10 $\mu$M, 30 $\mu$M, 50 $\mu$M, 80 $\mu$M and 150 $\mu$M) of compounds (**1**–**7** except for **4**), comparing to concentrations (0.0016 $\mu$M, 0.008 $\mu$M, 0.04 $\mu$M, 0.2 $\mu$M, 0.5 $\mu$M, 1 $\mu$M and 1.5 $\mu$M ) of tacrine as the positive control were evalued and each concentration was analyzed for three times. Compounds were dissolved in DMSO (0.1%). Inhibition curves were obtained by using origin 8 software and $IC_{50}$ values were obtained by plotting the percentage of inhibition versus the concentration.

**Anti-BuChE assay.** The Anti-BuChE assay was performed as previously described.

**Neurotrophic Bioassay.**[11] PC12 cells are bought from Kunming Institute of Zoology, Chinese Academy of Scienses and suspended in 12.5% HS + 2.5% FBS, then seeded at 50,000 cells/mL into poly-L-lysine-coated 48 well culture plates. After 48 h, the medium is changed to a serum-free medium F12 (10% HS + 5% FBS + 10 mg/mL NGF).[12] Samples at 50 $\mu$M and 5 $\mu$M are added into F12. The length of axon was measured and calculated by using microscope.

## Electronic Supplementary Material

Supplementary material is available in the online version of this article at http://dx.doi.org/ 10.1007/s13659-012-0026-z and is accessible for authorized users.

## Acknowledgments

This work was supported by the National Natural Science Foundation (20872148). The authors are grateful to the analytical group of the Laboratory of Phytochemistry, Kunming Institute of Botany, Chinese Academy of Sciences for measuring NMR, MS, and IR data.

**Open Access** This article is distributed under the terms of the Creative Commons Attribution License which permits any use, distribution, and reproduction in any medium, provided the original author(s) and source are credited.

## Electronic supplementary material

Supplementary material, approximately 4.75 MB.
