# Supplementary material for: Sesquilignans and sesquiterpenoid from the stem barks of Illicium simonsii and their anti-AChE activity
Source: Nat Prod Bioprospect. 2012 May 24;2(3):133–7. doi: 10.1007/s13659-012-0026-z (PMC4131593; doi:10.1007/s13659-012-0026-z)
Supplement: Supplementary file 1 — Supplementary material, approximately 4.75 MB. [file 13659_2012_26_MOESM1_ESM.pdf]

**Electronic Supplementary Material**

# Sesquilignans and sesquiterpenoid from the stem barks of *Illicium simonsii* and their anti-AChE activity

Chuan-Fu DONG,[a,b] Lei LIU,[a,b] Huai-Rong LUO,[a] Xiao-Nian LI,[a] Zheng-Ye GUAN,[a,b] and Yi-Fen WANG[a,*]

[a]State Key Laboratory of Phytochemistry and Plant Resources in West China, Kunming Institute of Botany, Chinese Academy of Sciences, Kunming 650201, China

[b]Graduate University of Chinese Academy of Sciences, Beijing 100049, China

Received 25 March 2012; Accepted 16 May 2012

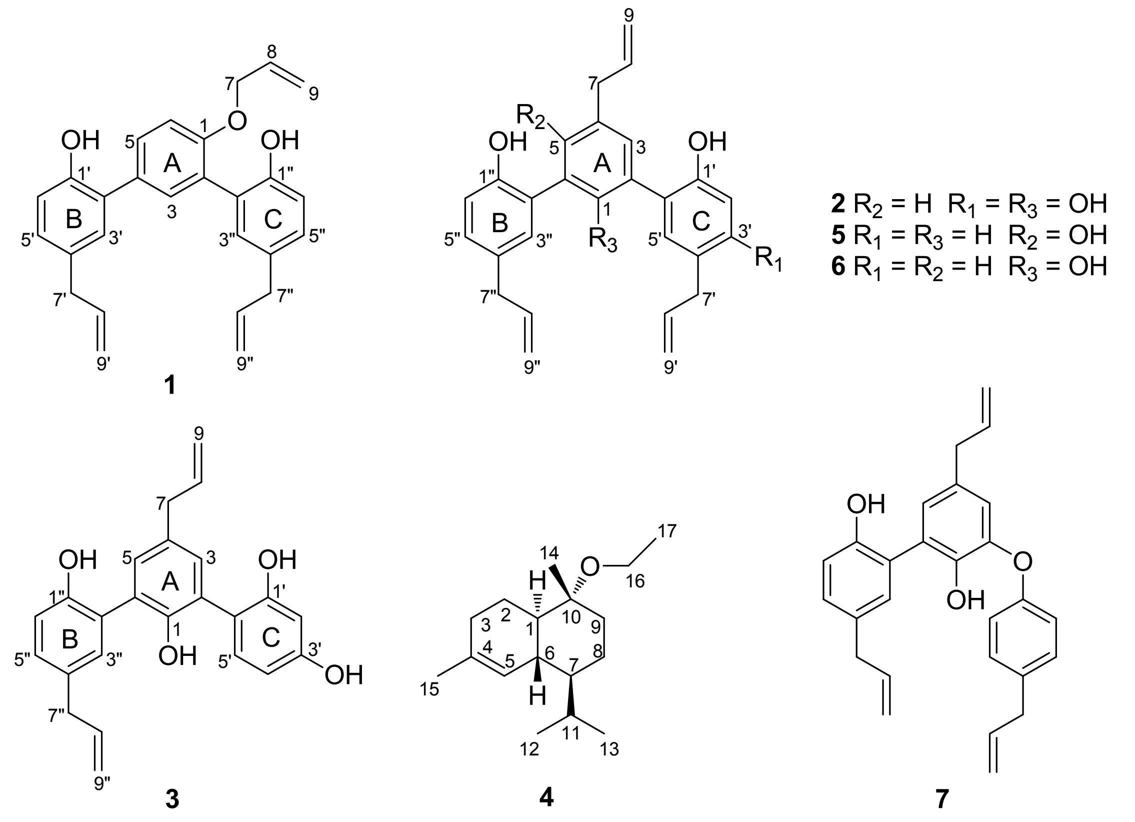


Structures of compounds **1**–**7**

*To whom correspondence should be addressed. E-mail: wangyifen@mail.kib.ac.cn

## Supporting Information

Figure 1S-7S. NMR and MS spectra of compound **1**

Figure 8S-14S. NMR and MS spectra of compound **2**

Figure 15S-21S. NMR and MS spectra of compound **3**

Figure 22S-28S. NMR and MS spectra of compound **4**

Figure 1S. $^{1}$H NMR of compound **1**.

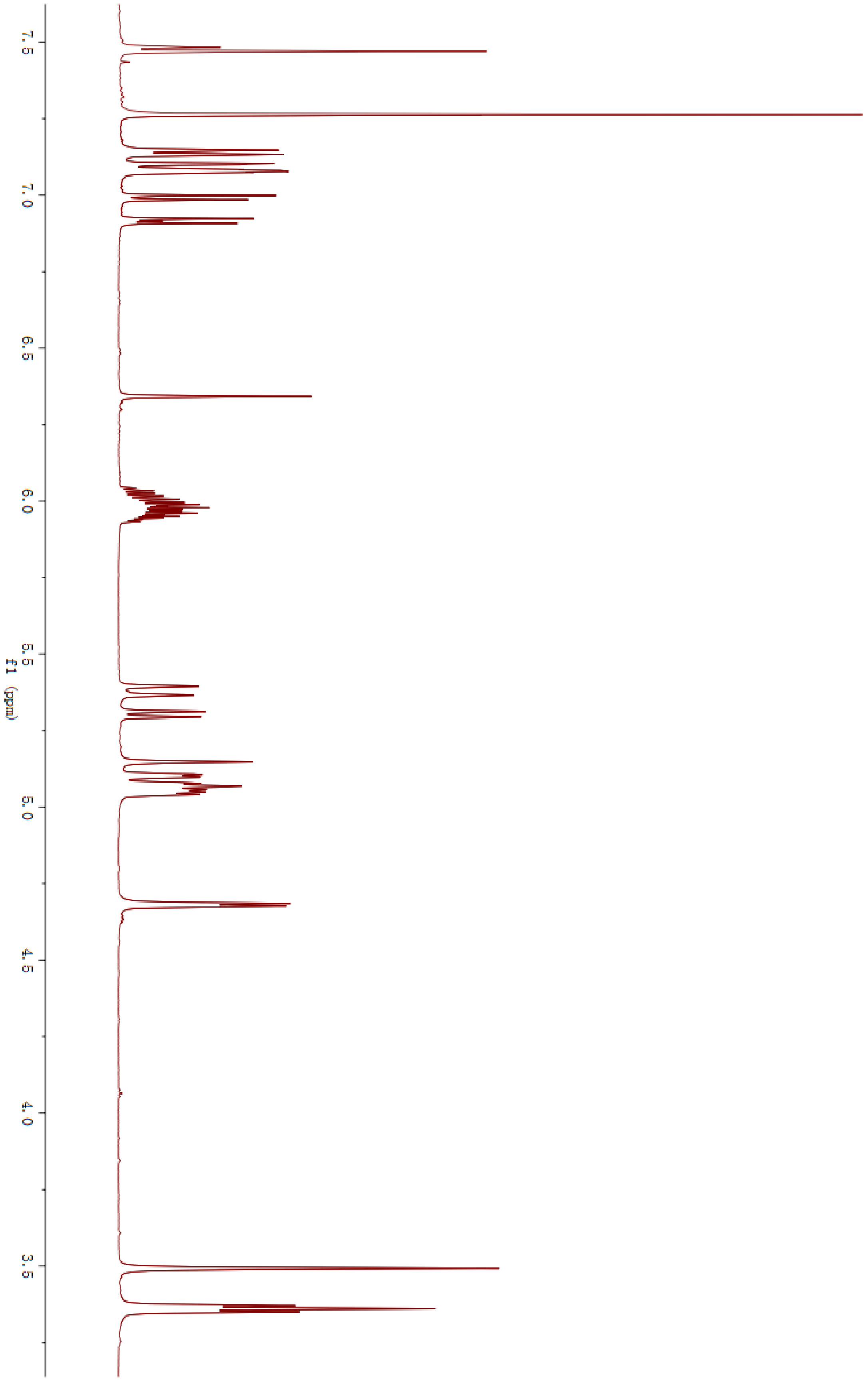

Figure 2S. $^{13}C$ NMR and DEPT of compound **1**.

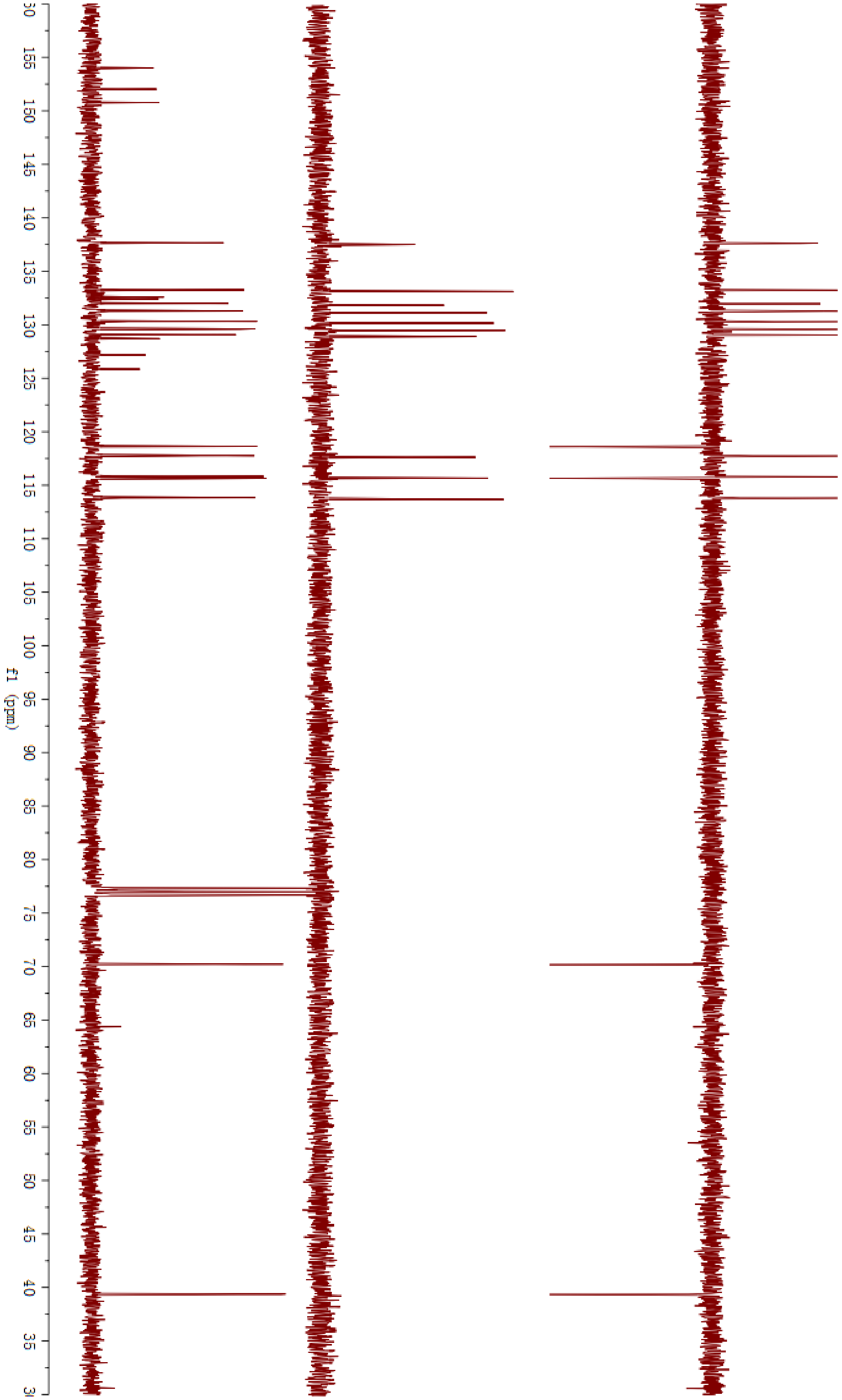

Figure 3S. HSQC of compound **1**.

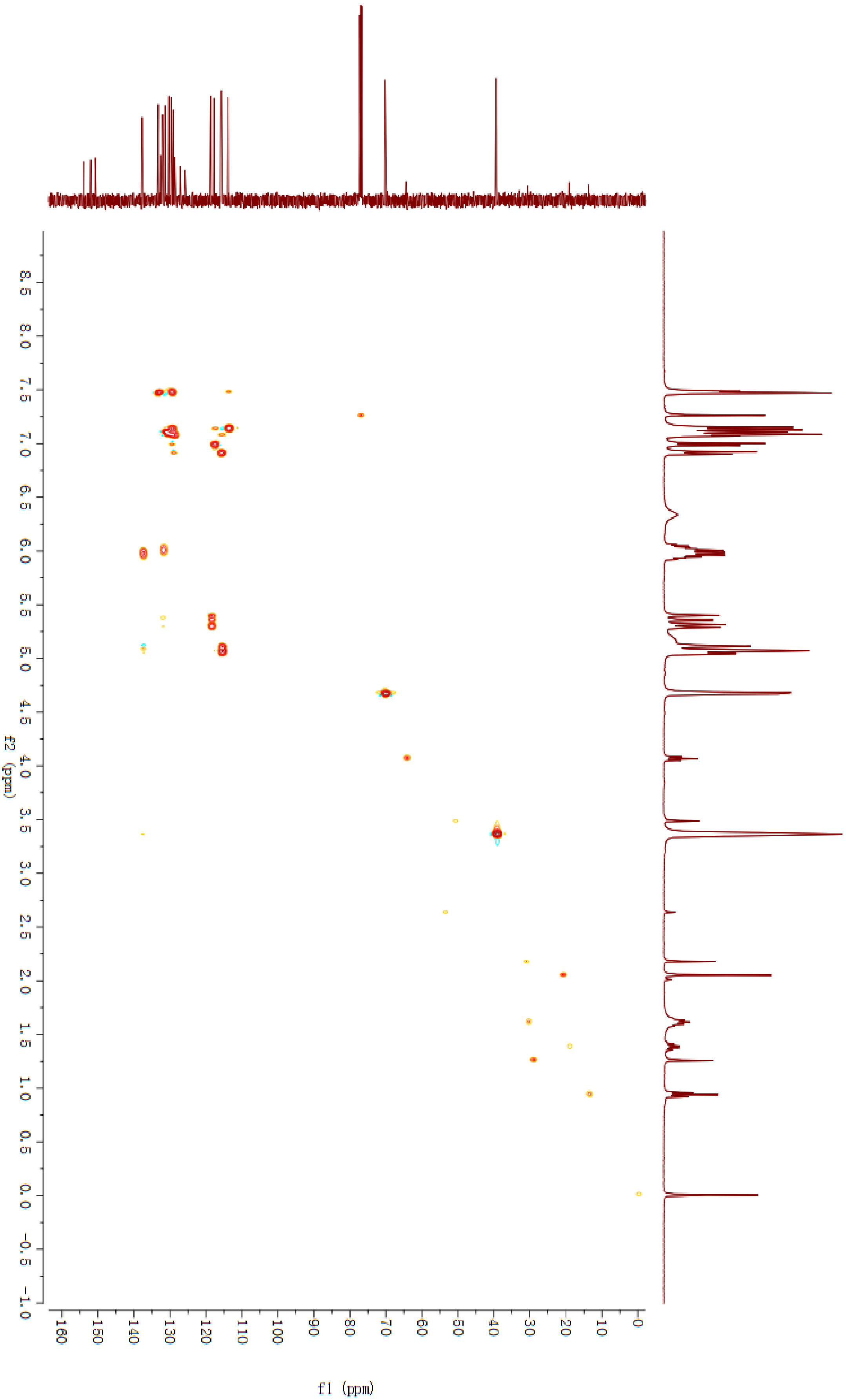

Figure 4S. HMBC of compound **1**.

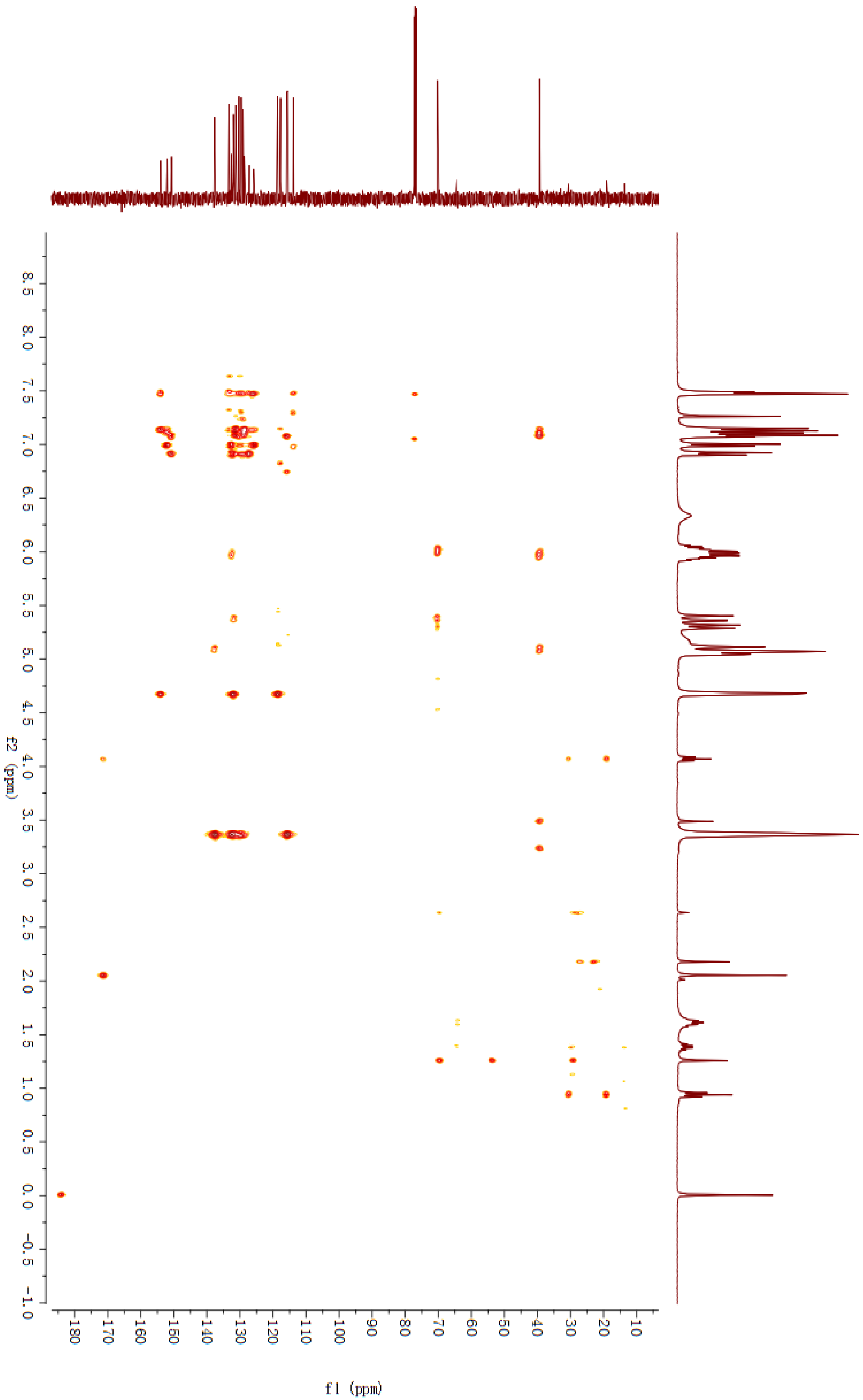

Figure 5S. H-H COSY of compound **1**.

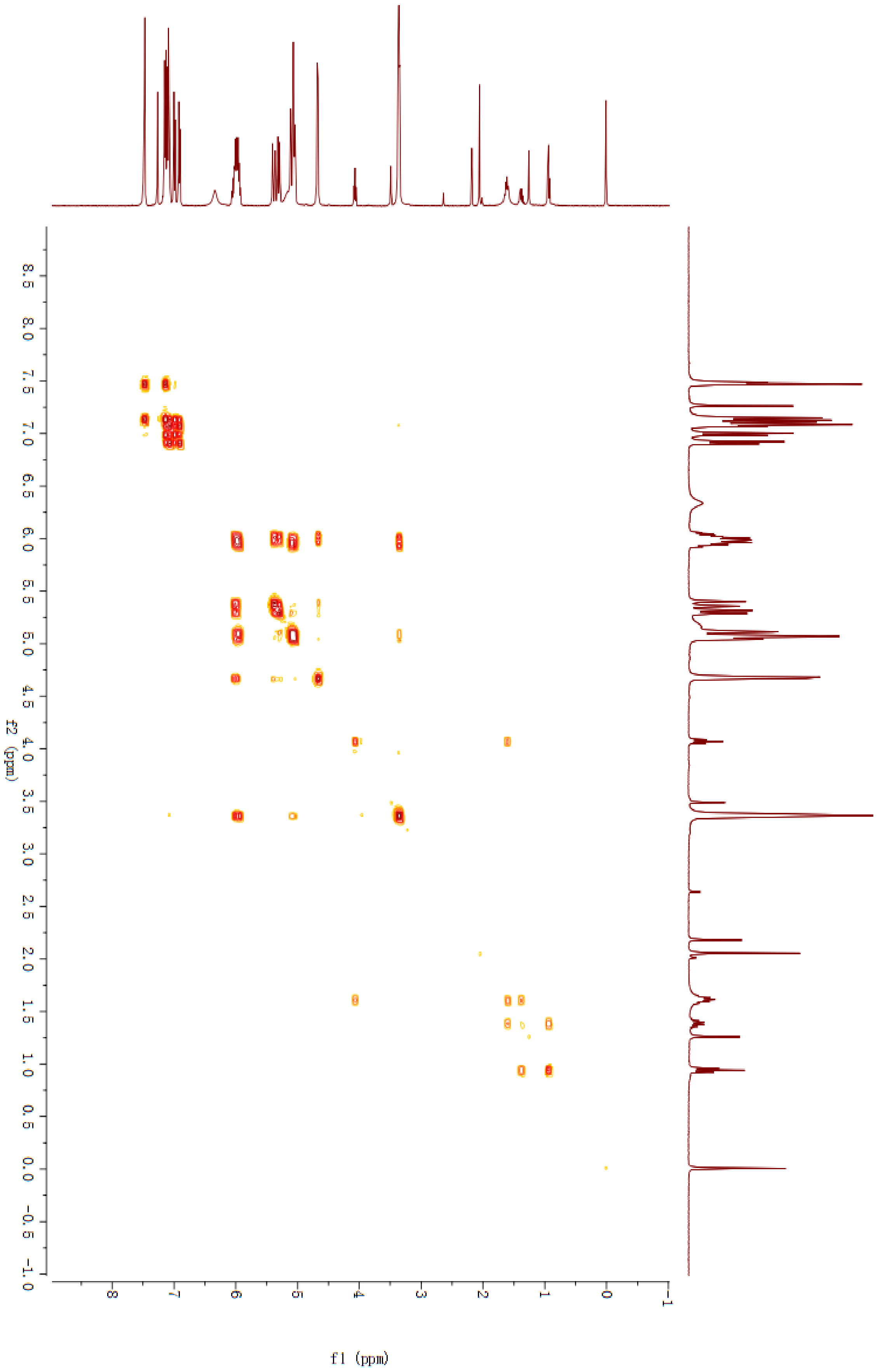

Figure 6S. ROESY of compound **1**.

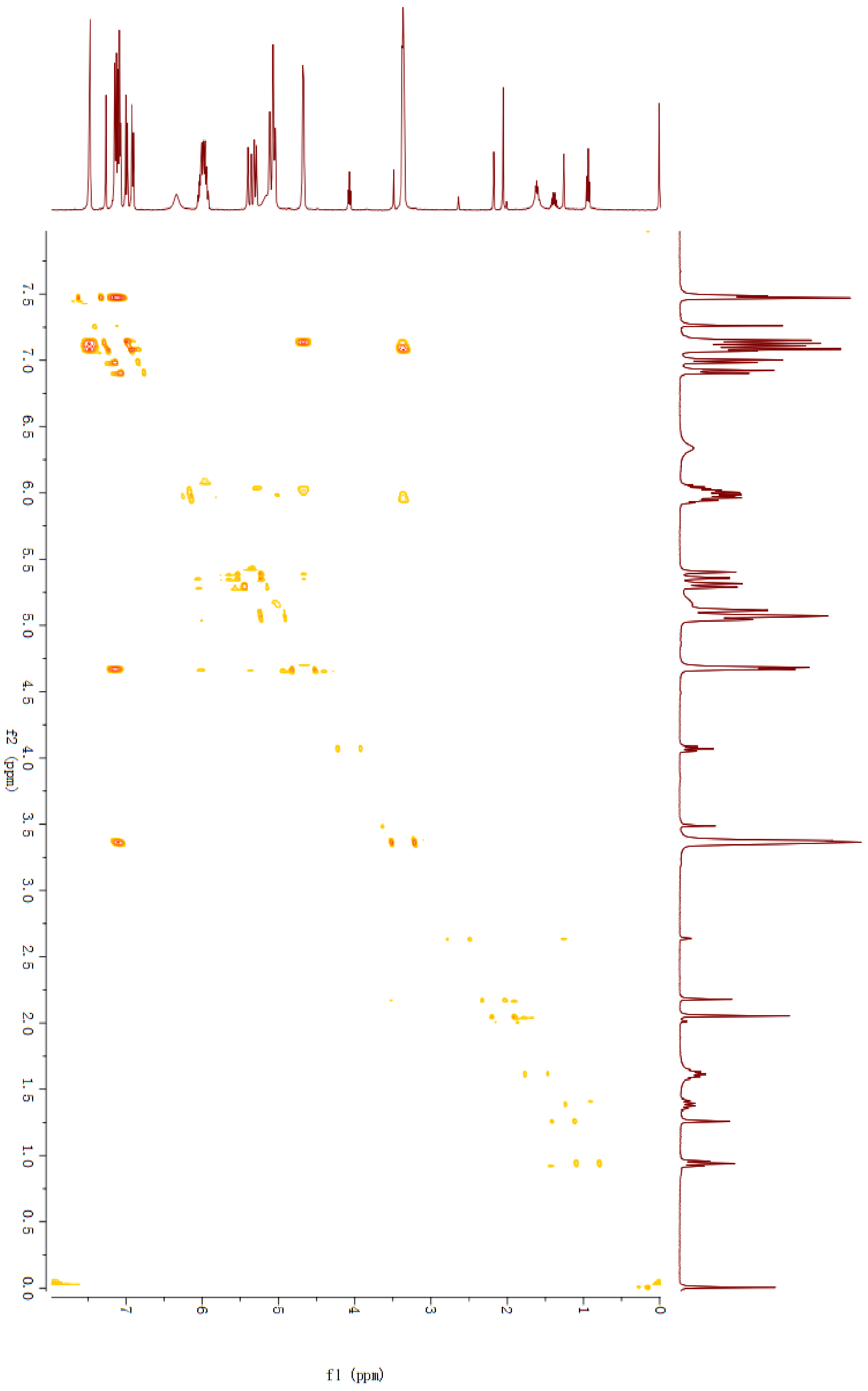

Figure 7S. HRESIMS of compound **1**.

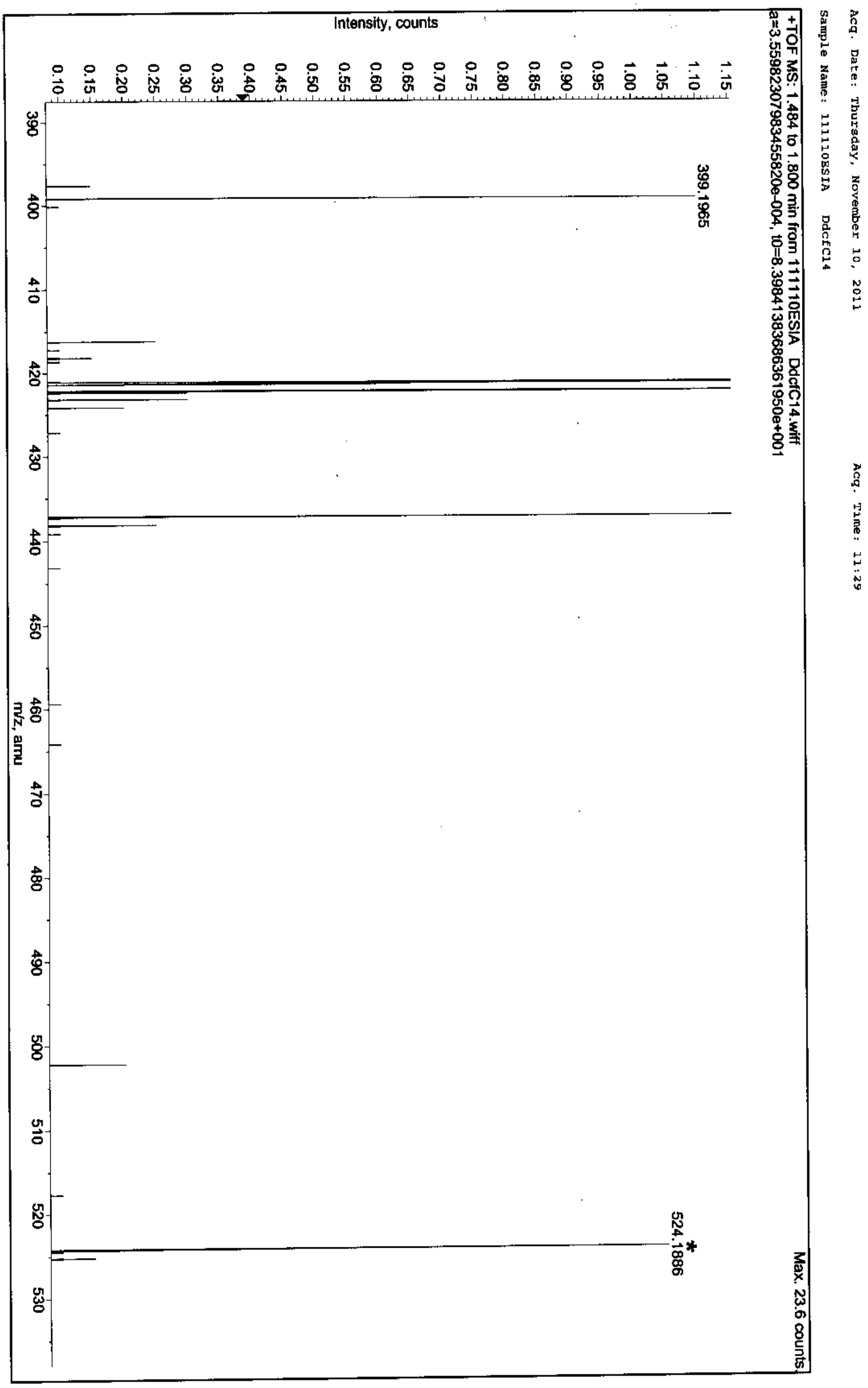

Figure 8S. $^{1}H$ NMR of compound **2**.

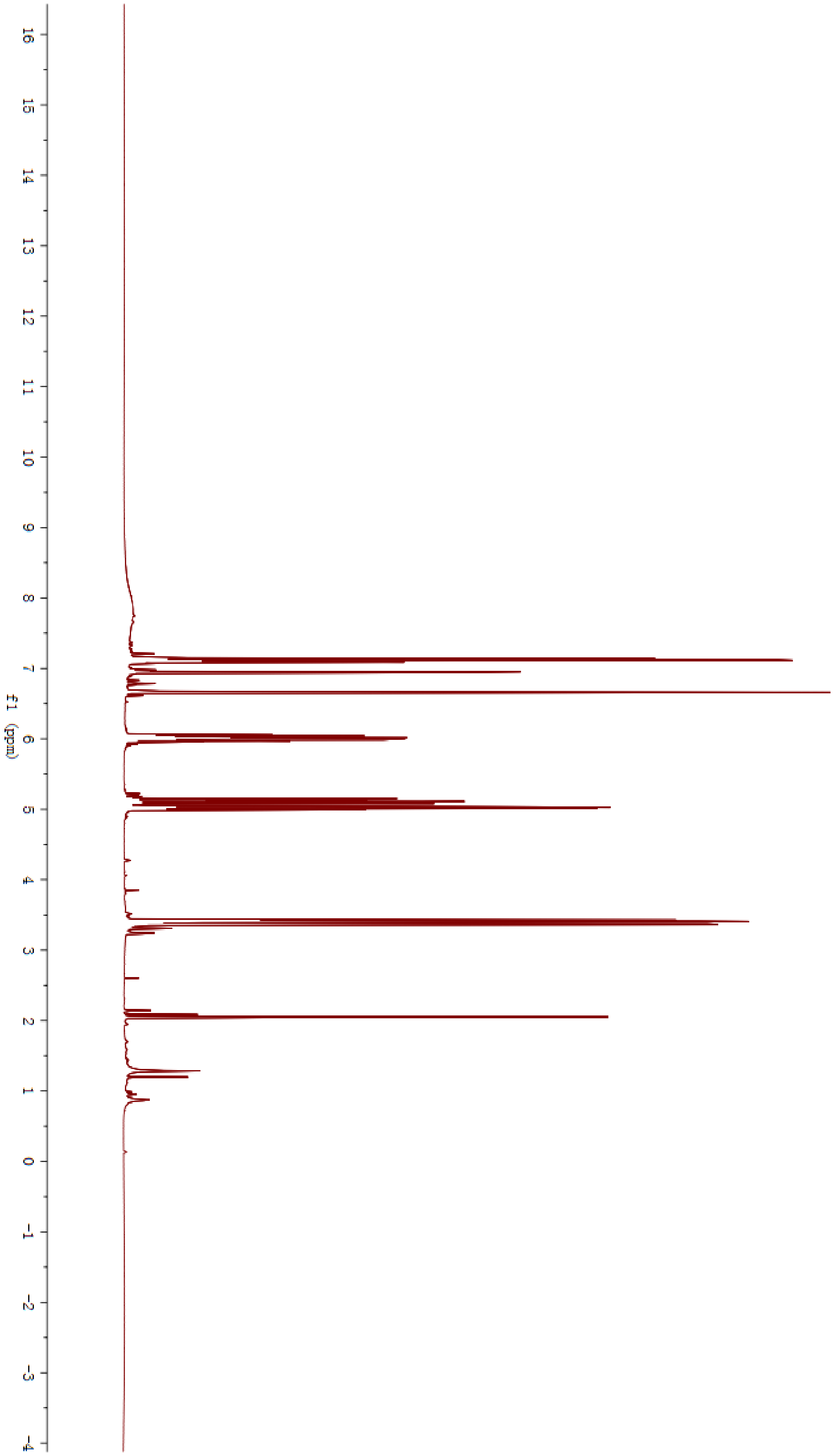


Figure 9S. $^{13}C$ NMR and DEPT of compound **2**.

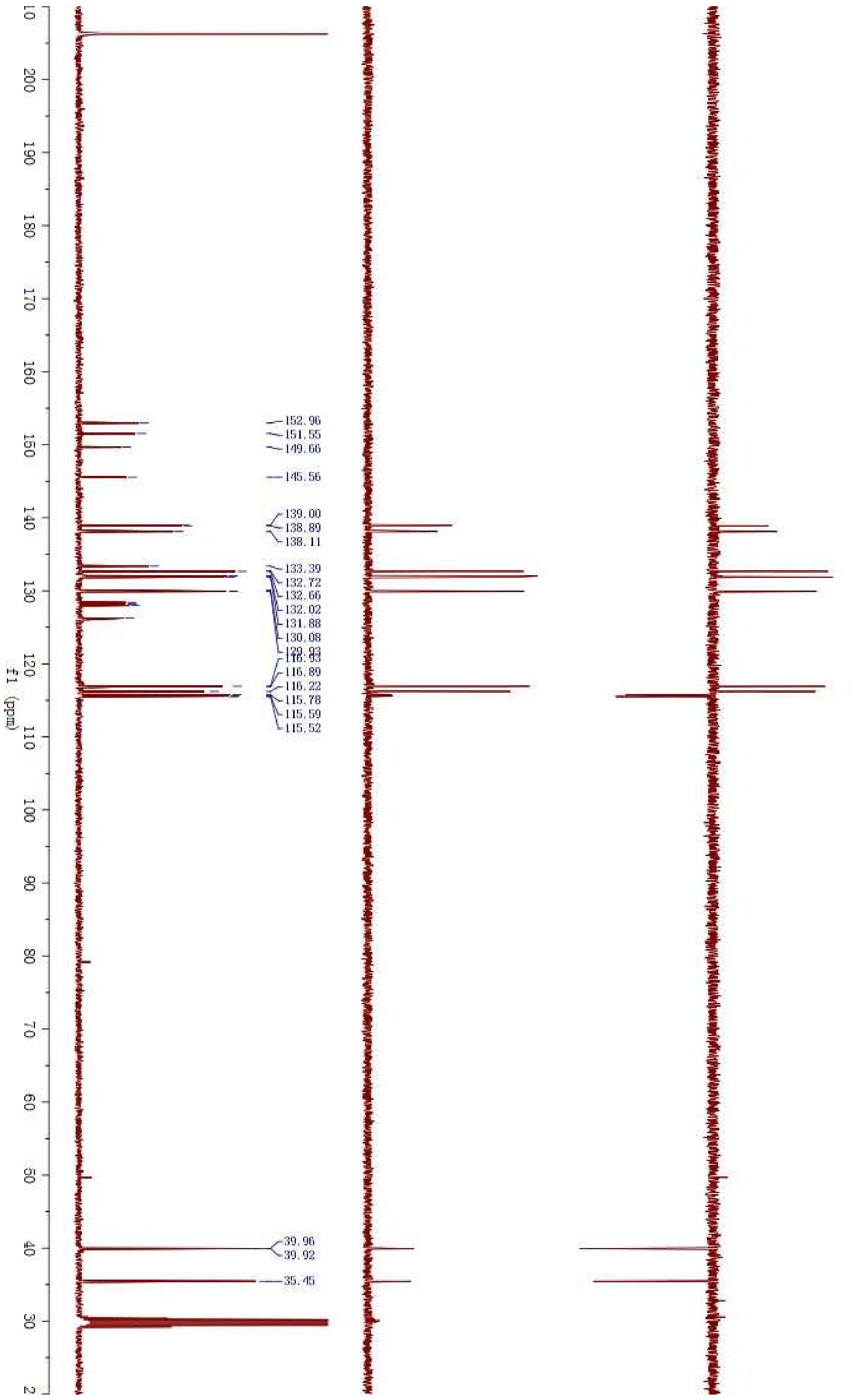


Figure 10S.

HSQC of compound **2**.

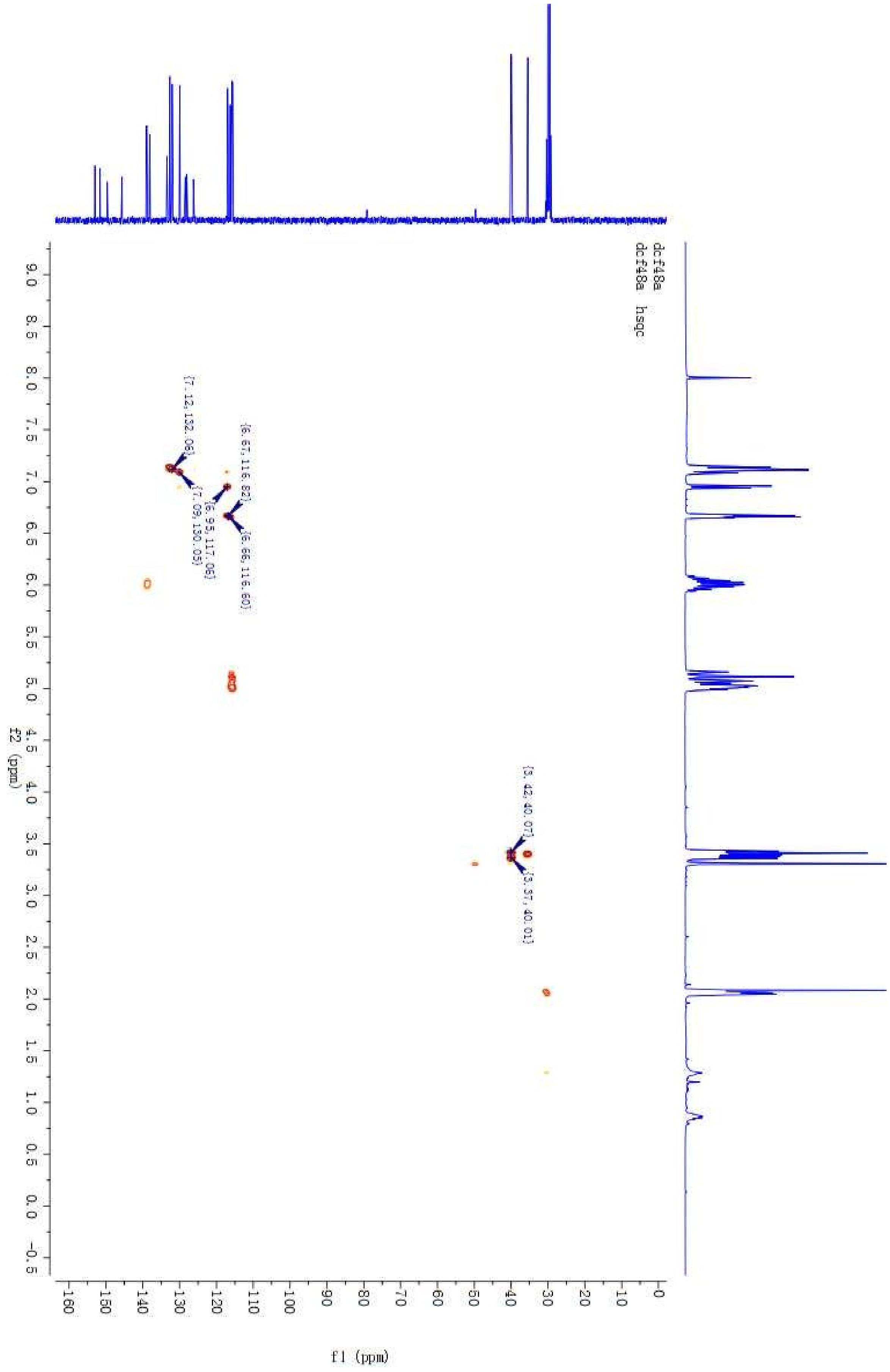


Figure 11S. HMBC of compound **2**.

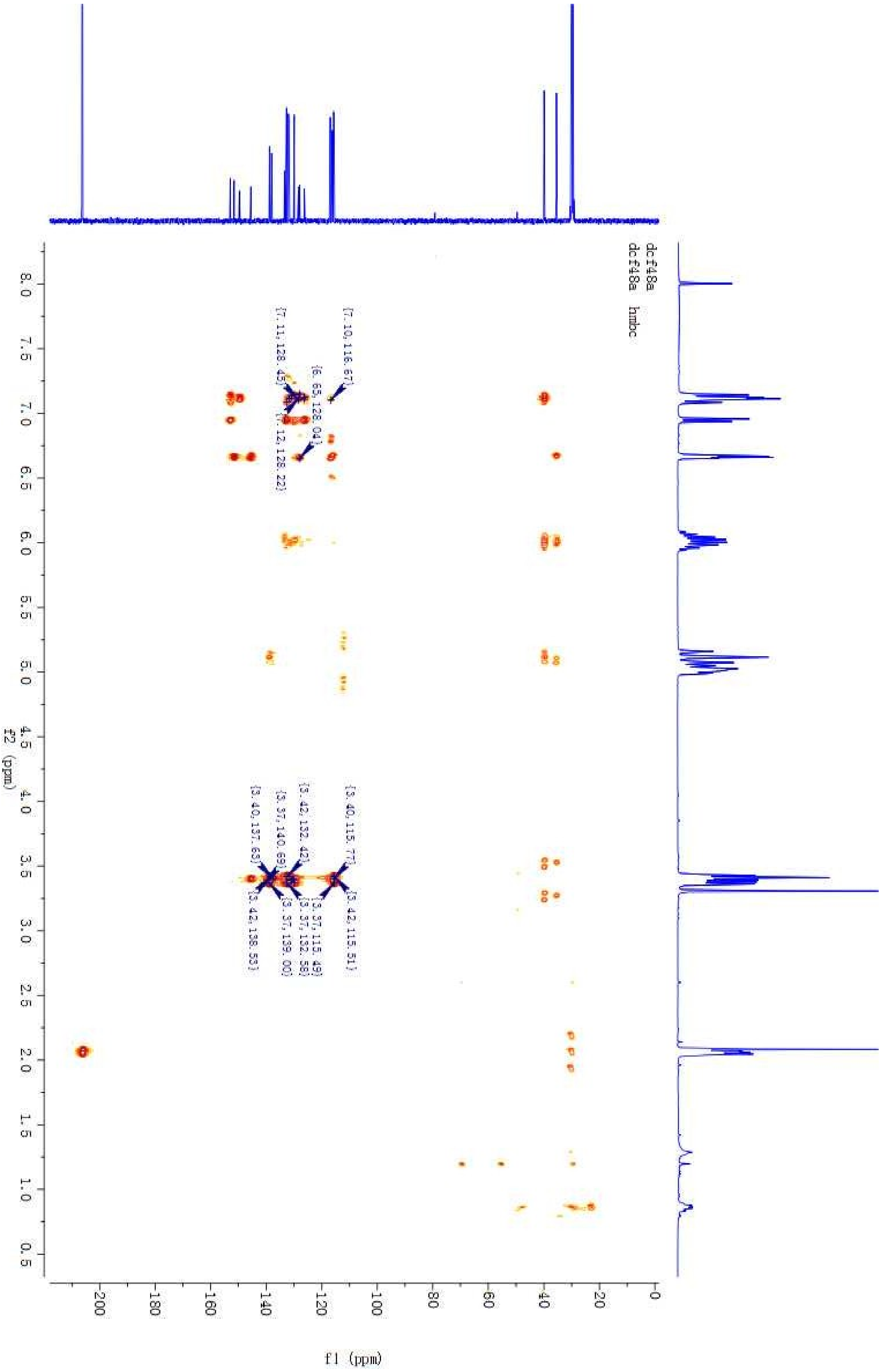


Figure 12S. $^{1}H$-$^{1}H$ COSY of compound **2**.

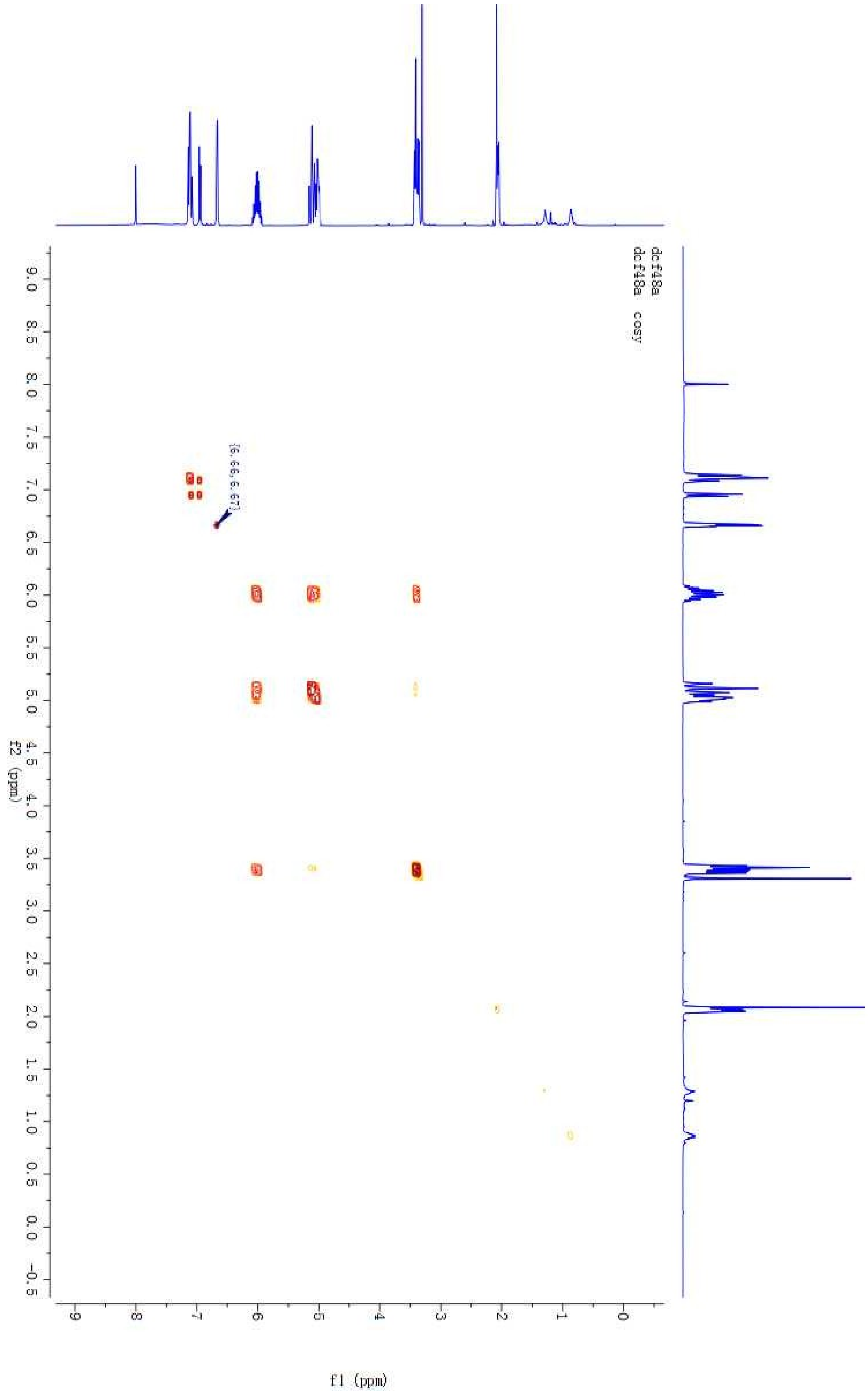


Figure 13S. ROESY of compound **2**.

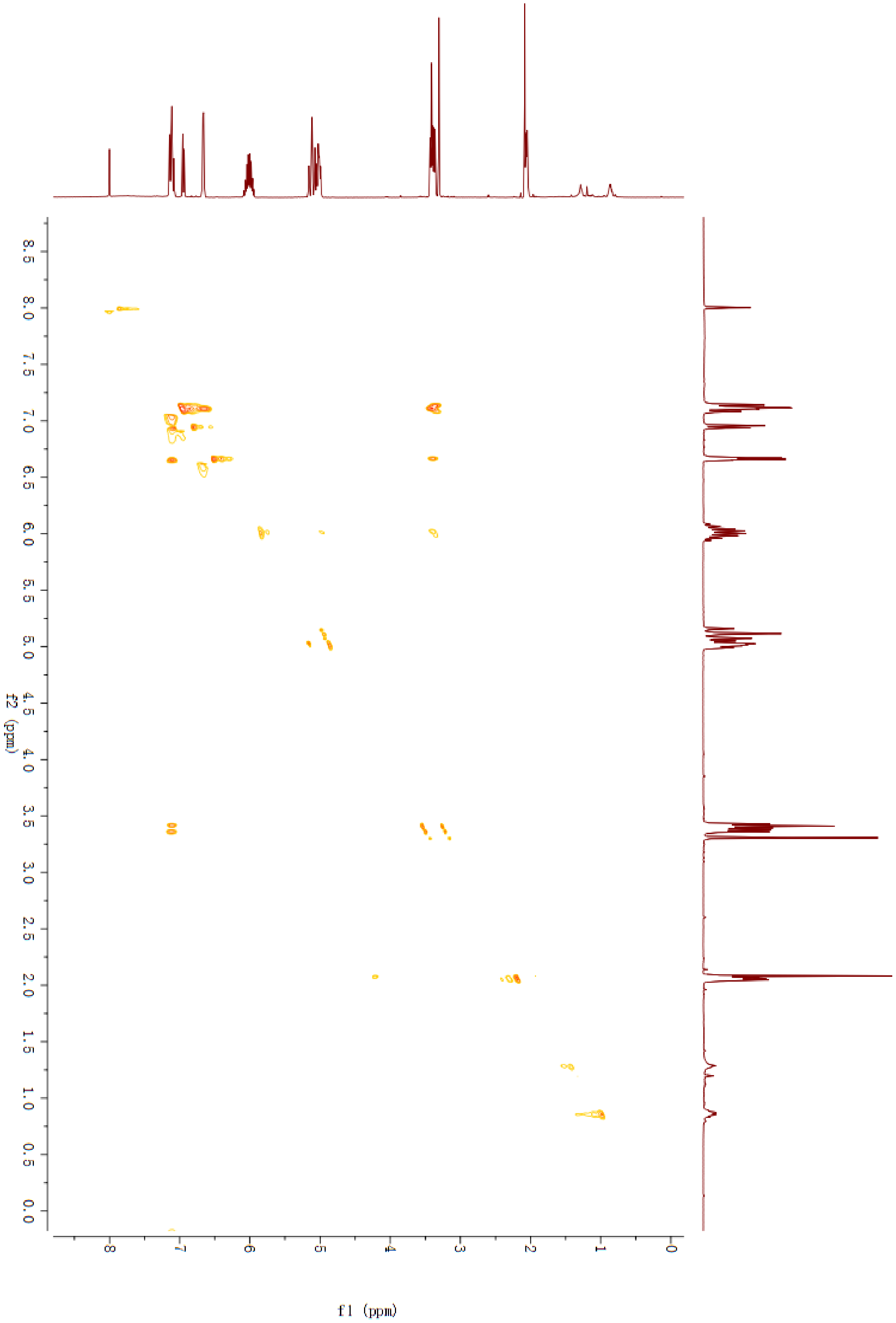


Figure 14S. HRESIMS of compound **2**.

Acq. Date: Monday, June 20, 2011    Acq. Time: 12:17

Sample Name: 110621ESIA  Ddcf48a

+TOF MS: 0.117 to 0.433 min from Sample 2 (110621ESIA  Ddcf48a) of 110621ESIA  Ddcf48a.wiff
a=3.56005755082081720e-004, t0=8.30885451465946970e+001

Max. 1877.8 counts.

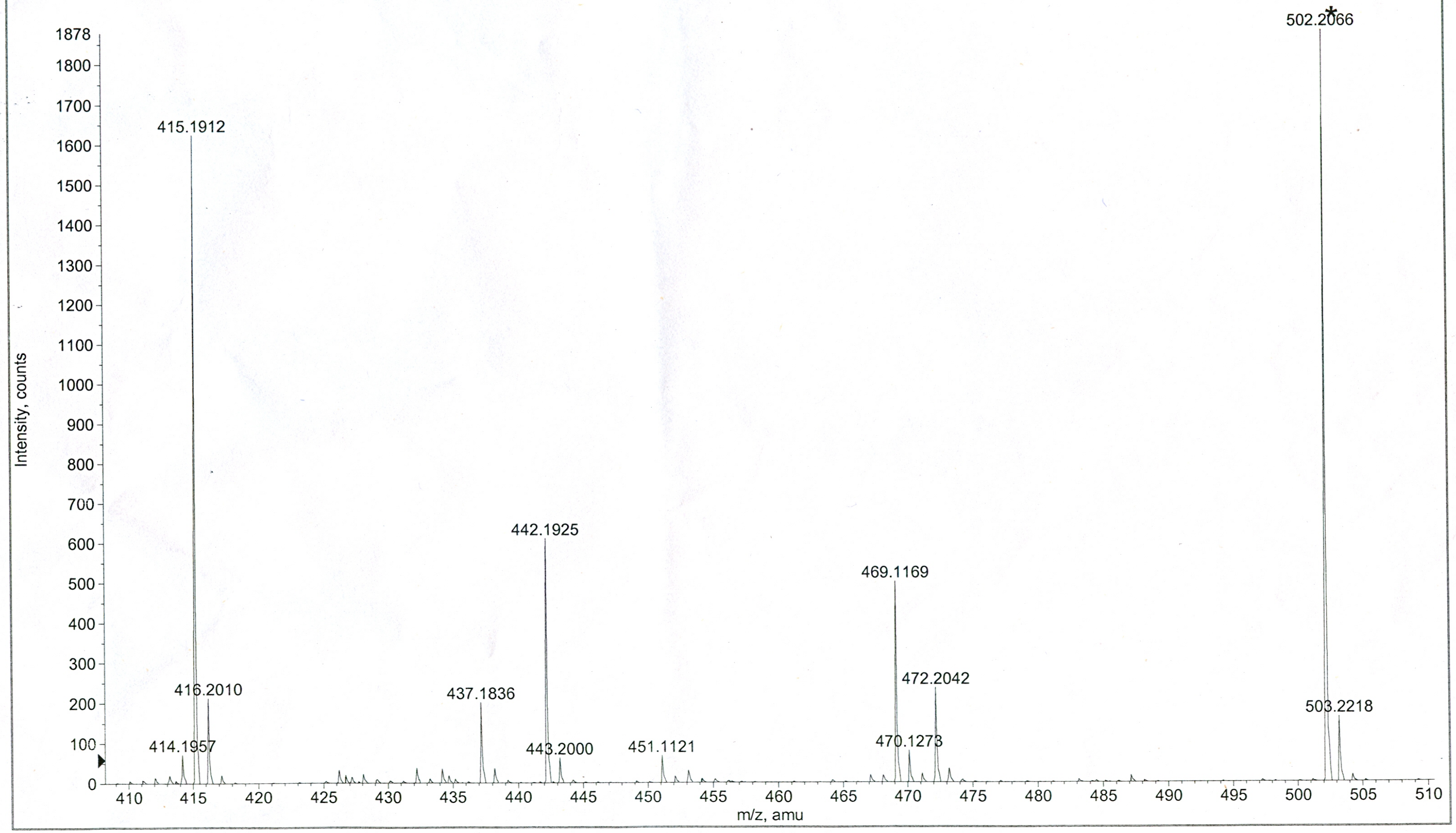

Figure 15S. $^{1}H$ NMR of compound **3**.

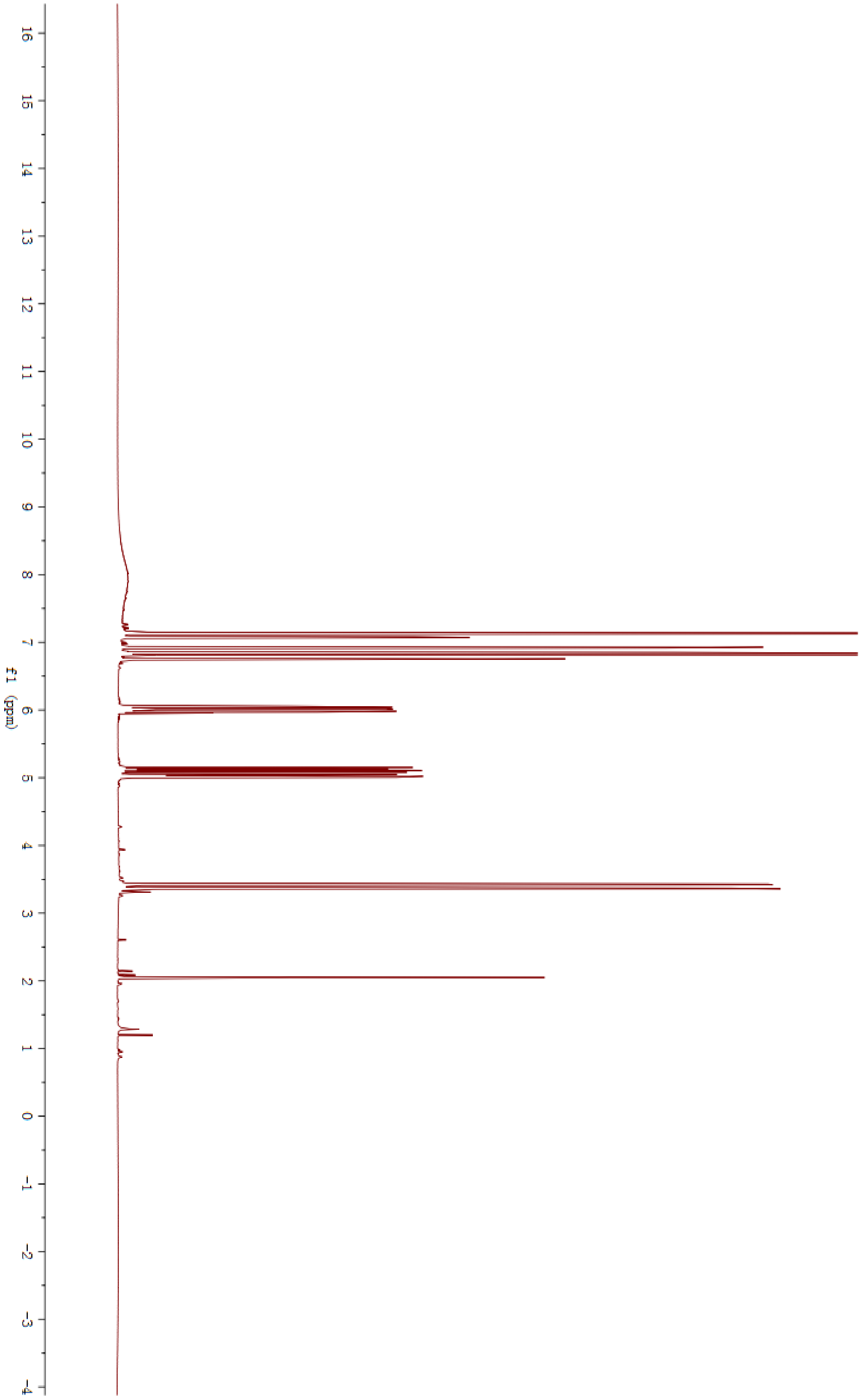

Figure 16S. $^{13}C$ NMR and DEPT of compound **3**.

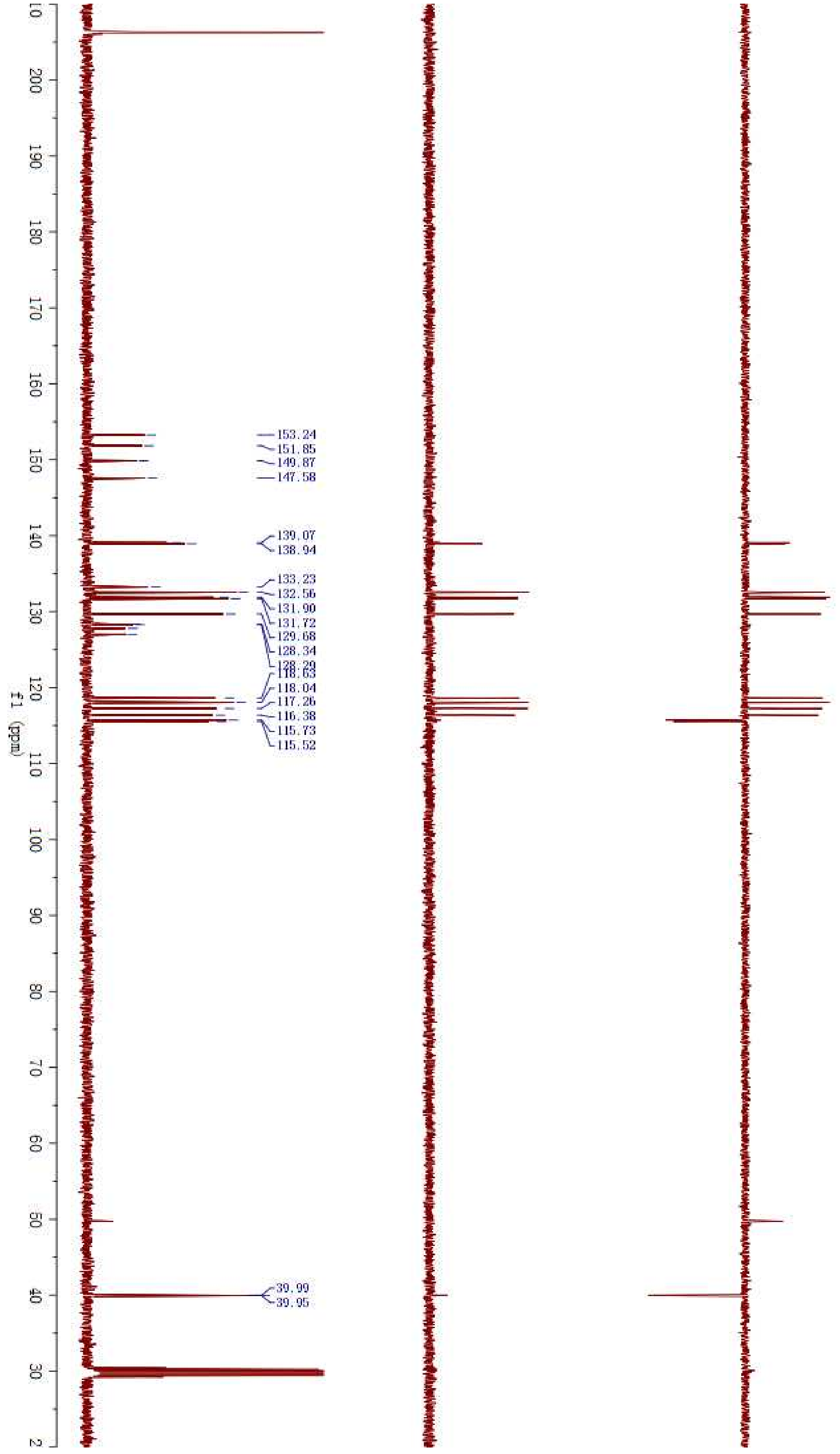

Figure 17S. HSQC of compound **3**.

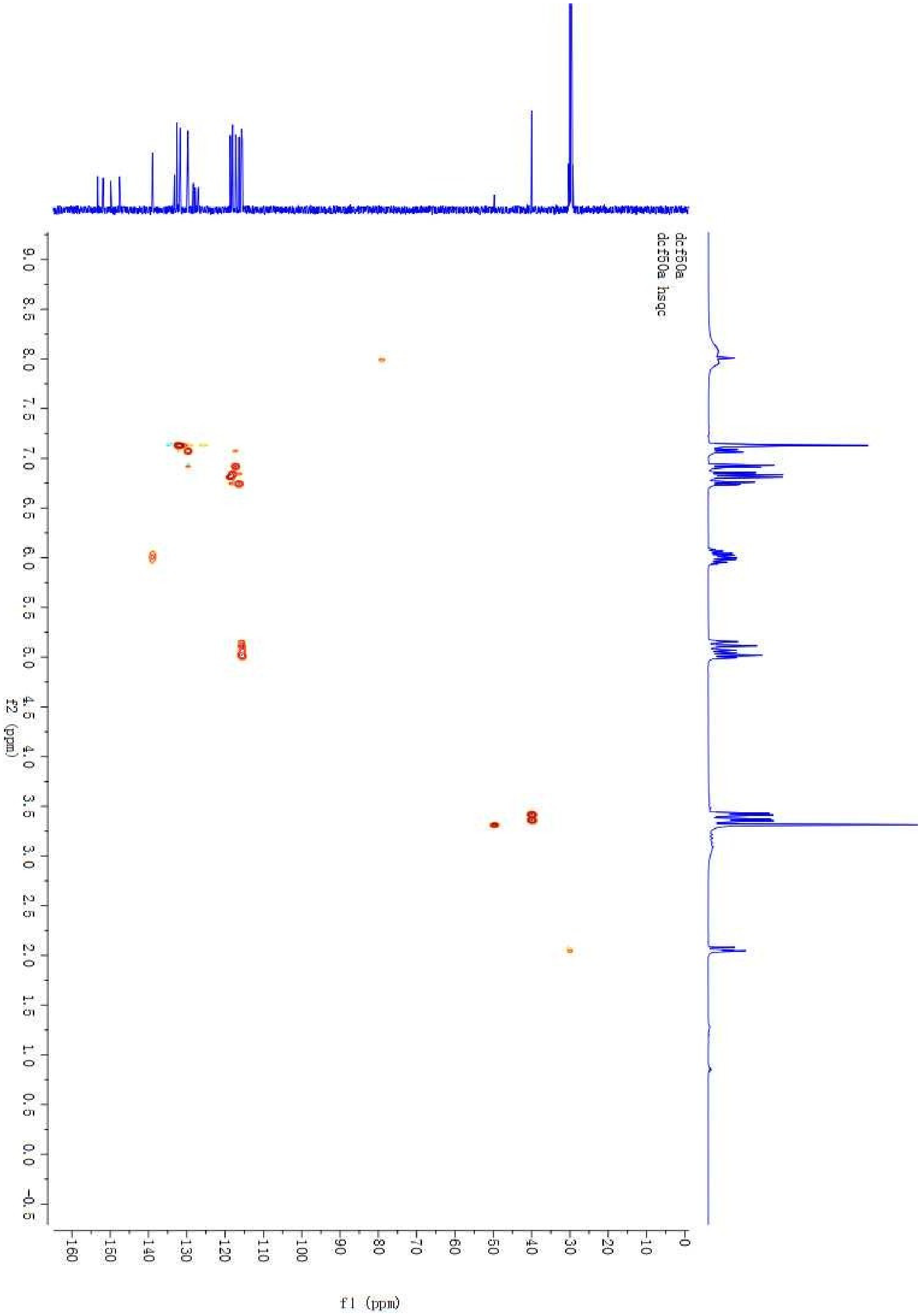

Figure 18S. HMBC of compound **3**.

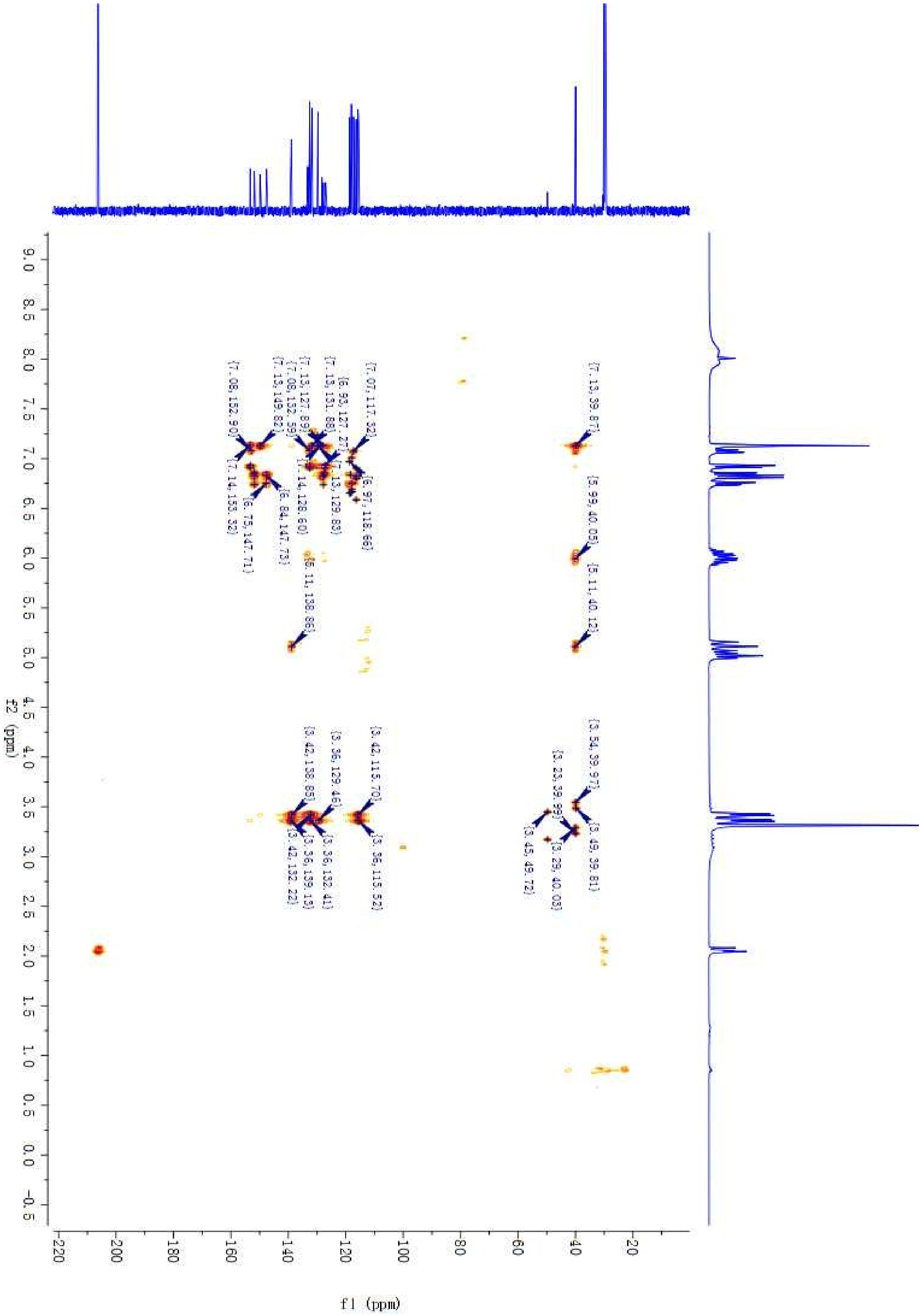

Figure 19S. H-H COSY of compound **3**.

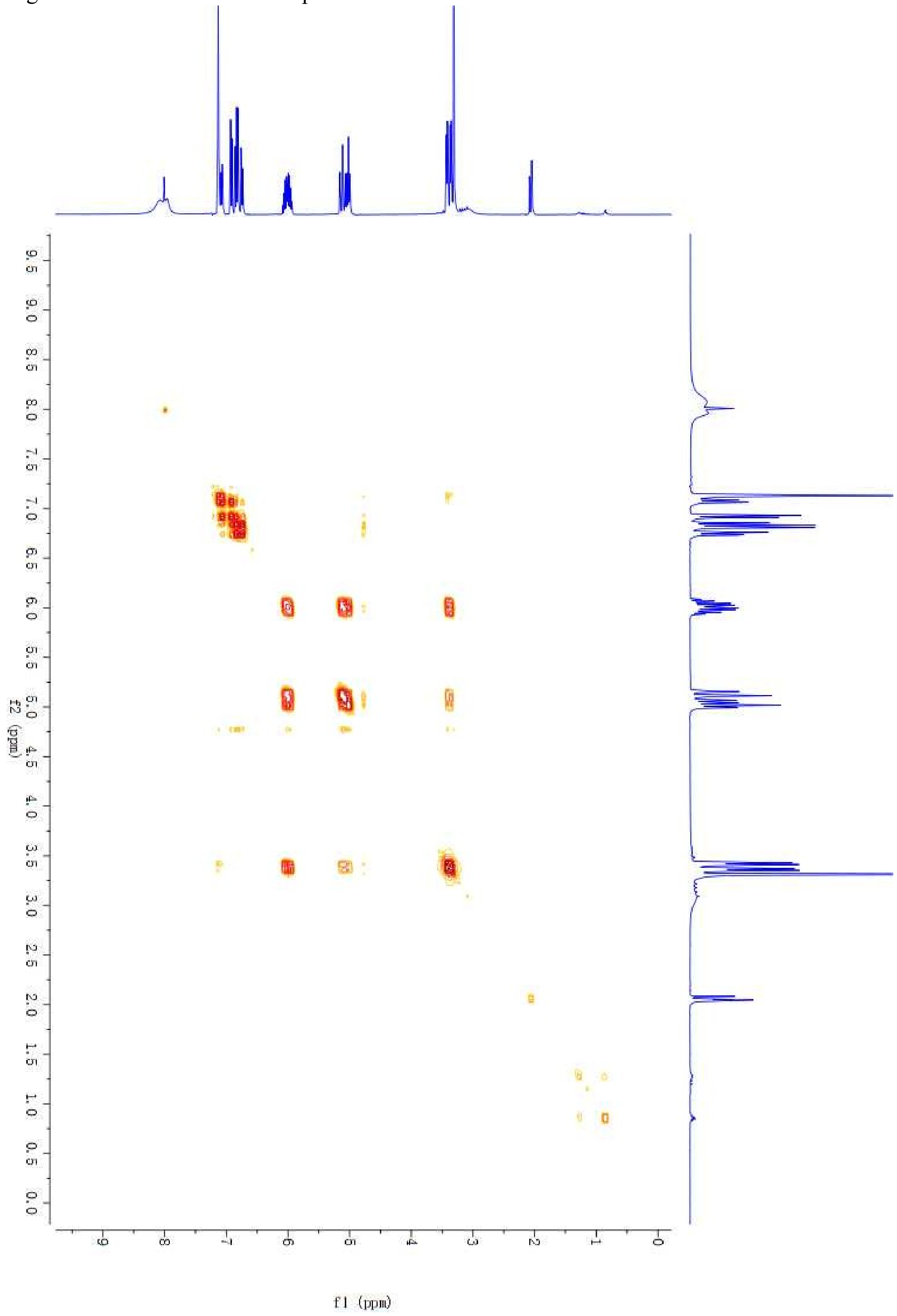

Figure 20S. ROESY of compound **3**.

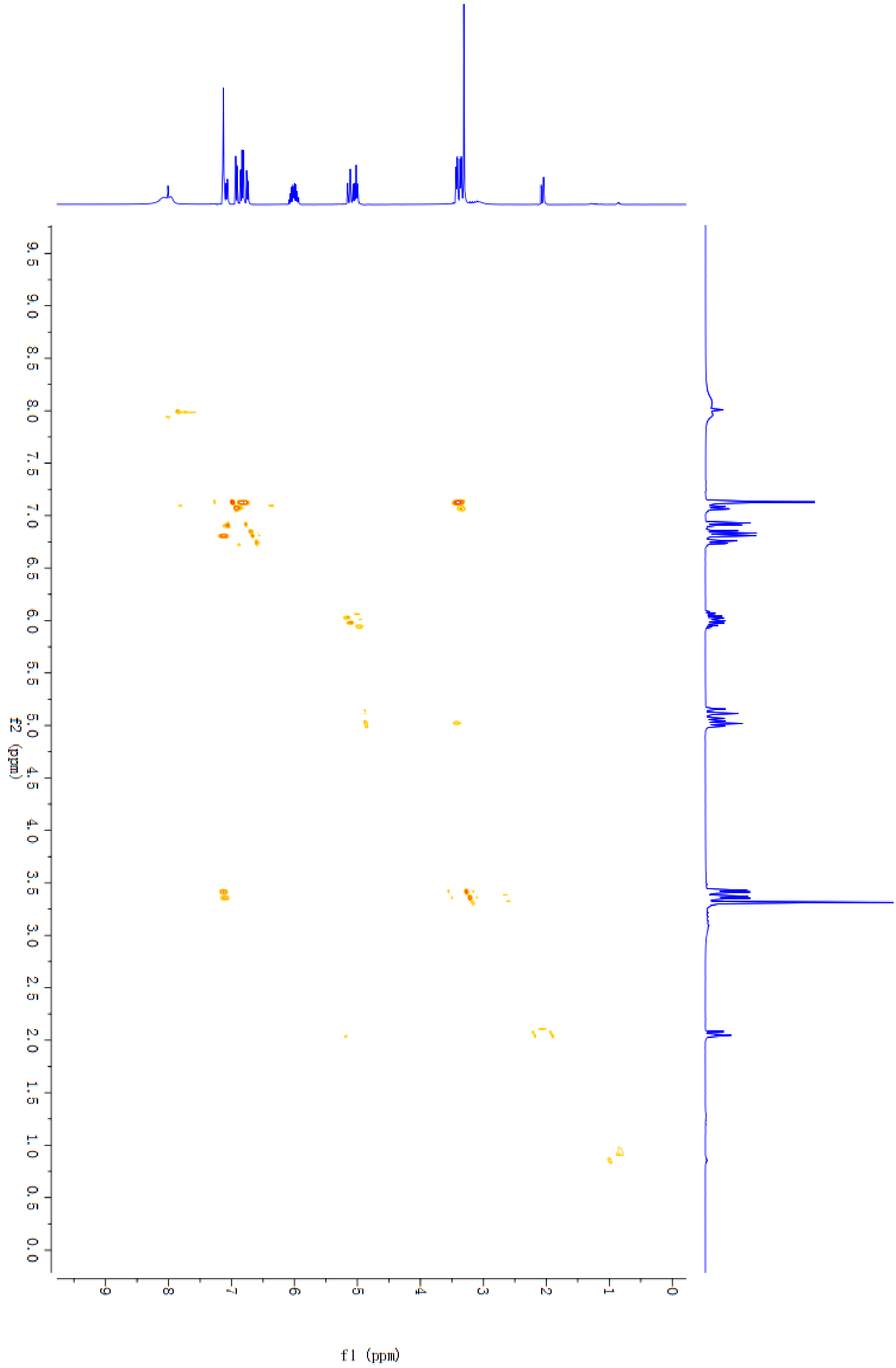

Figure 21S. HRESIMS of compound **3**.

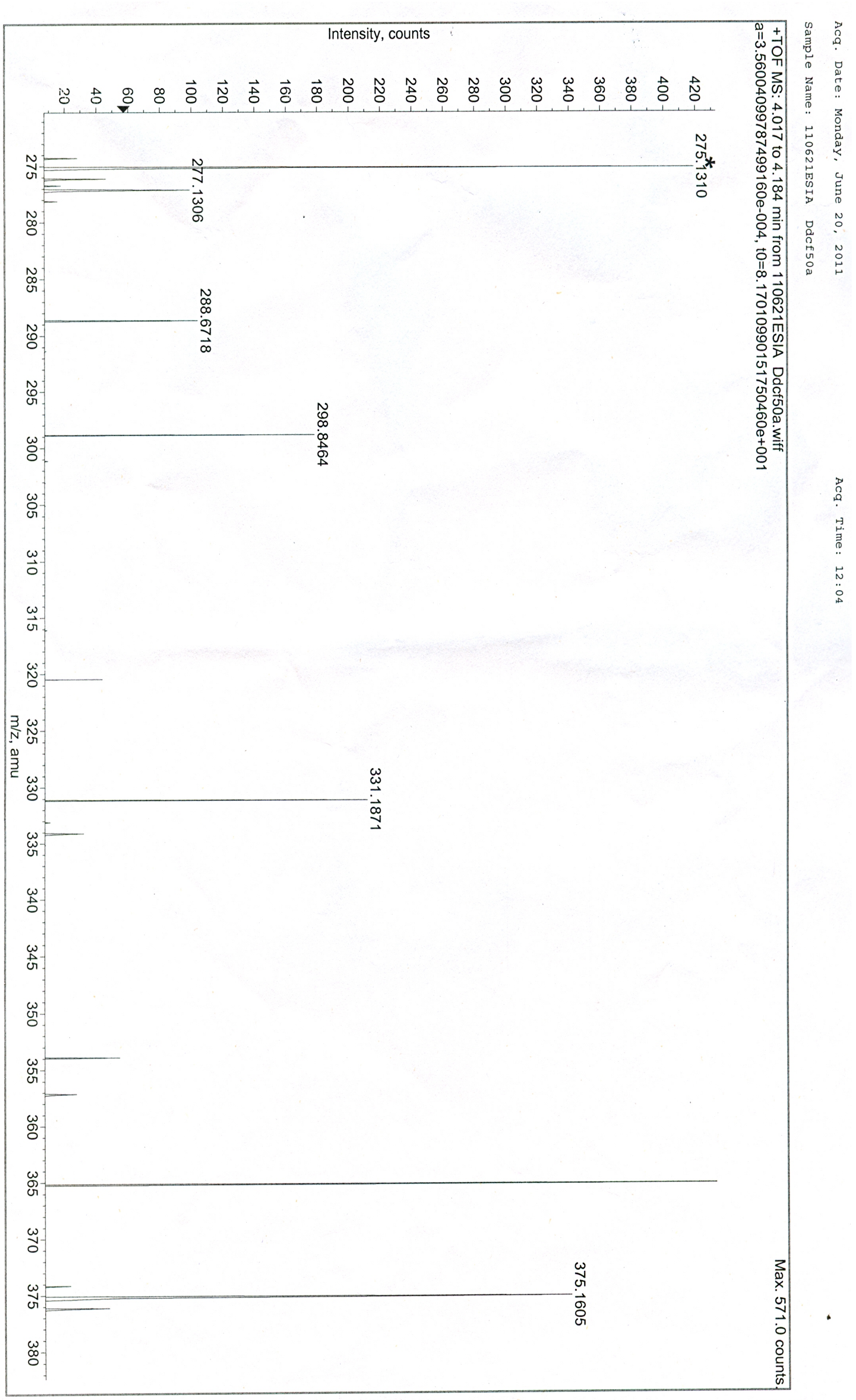

Figure 22S. $^{1}H$ NMR of compound **4**.

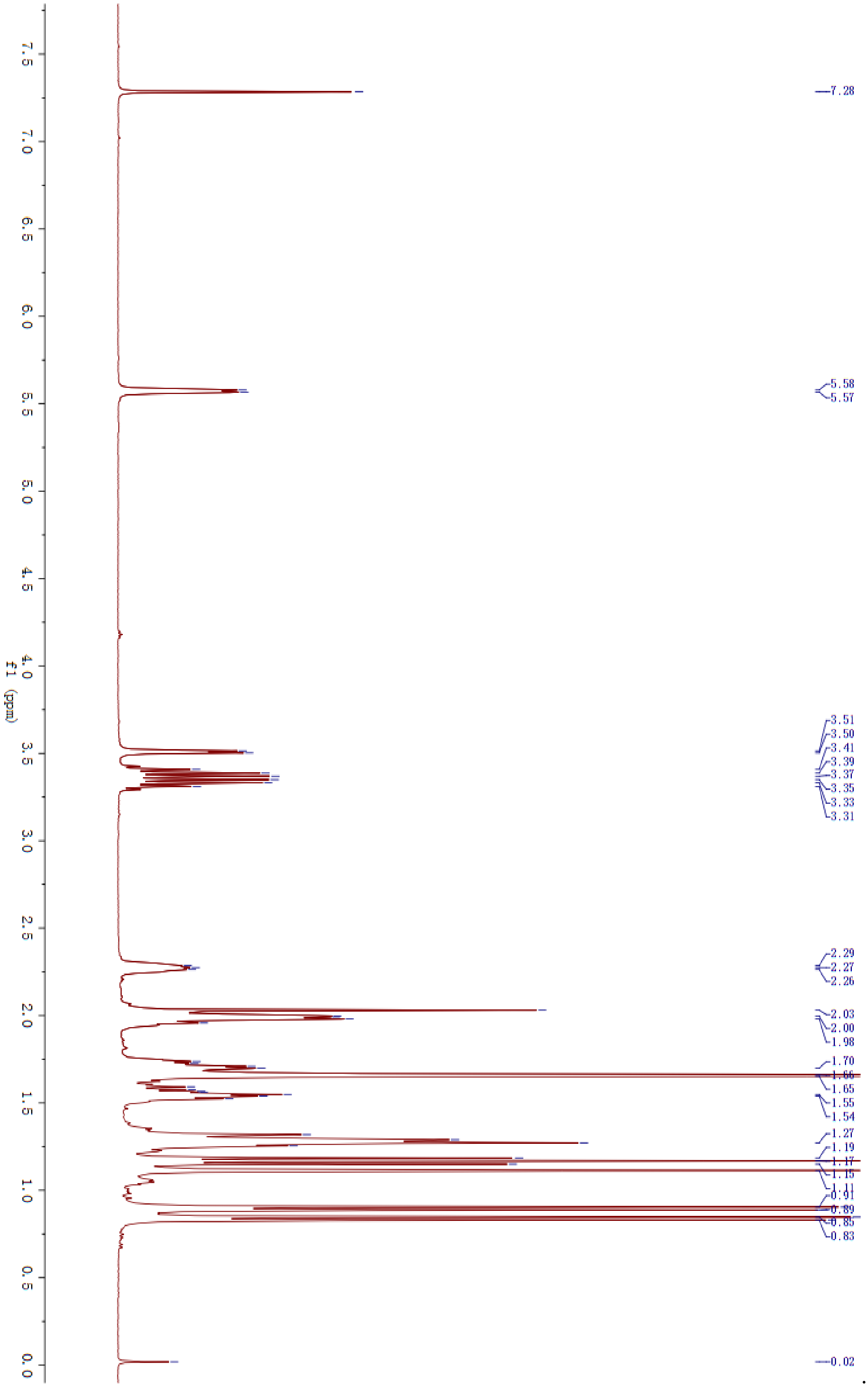


. Figure 23S.

$^{13}C$ NMR and DEPT of compound **4**.

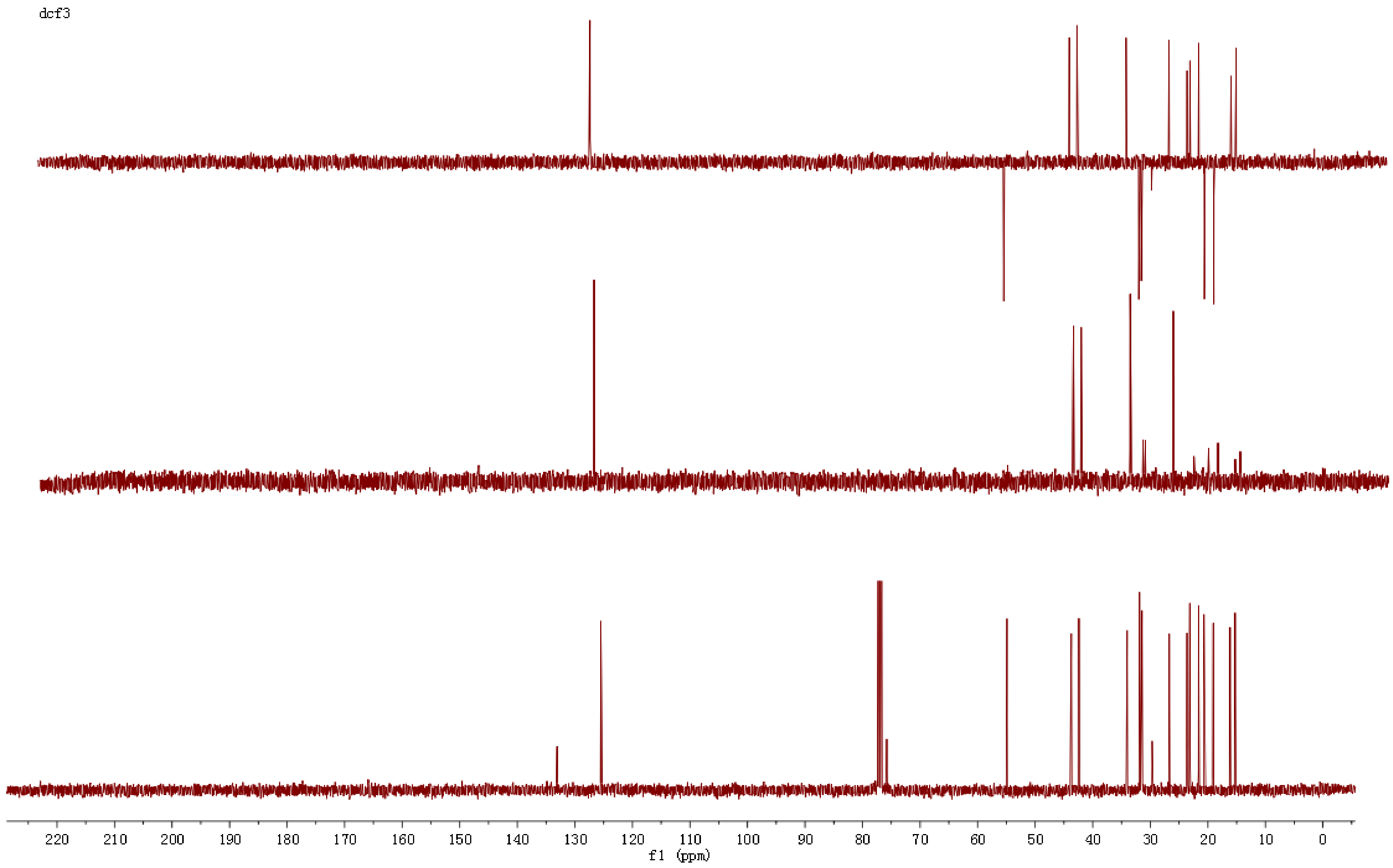
dcf3
220
210
200
190
180
170
160
150
140
130
120
110
100
90
80
70
60
50
40
30
20
10
0
f1 (ppm)

Figure 24S. HSQC of compound **4**.

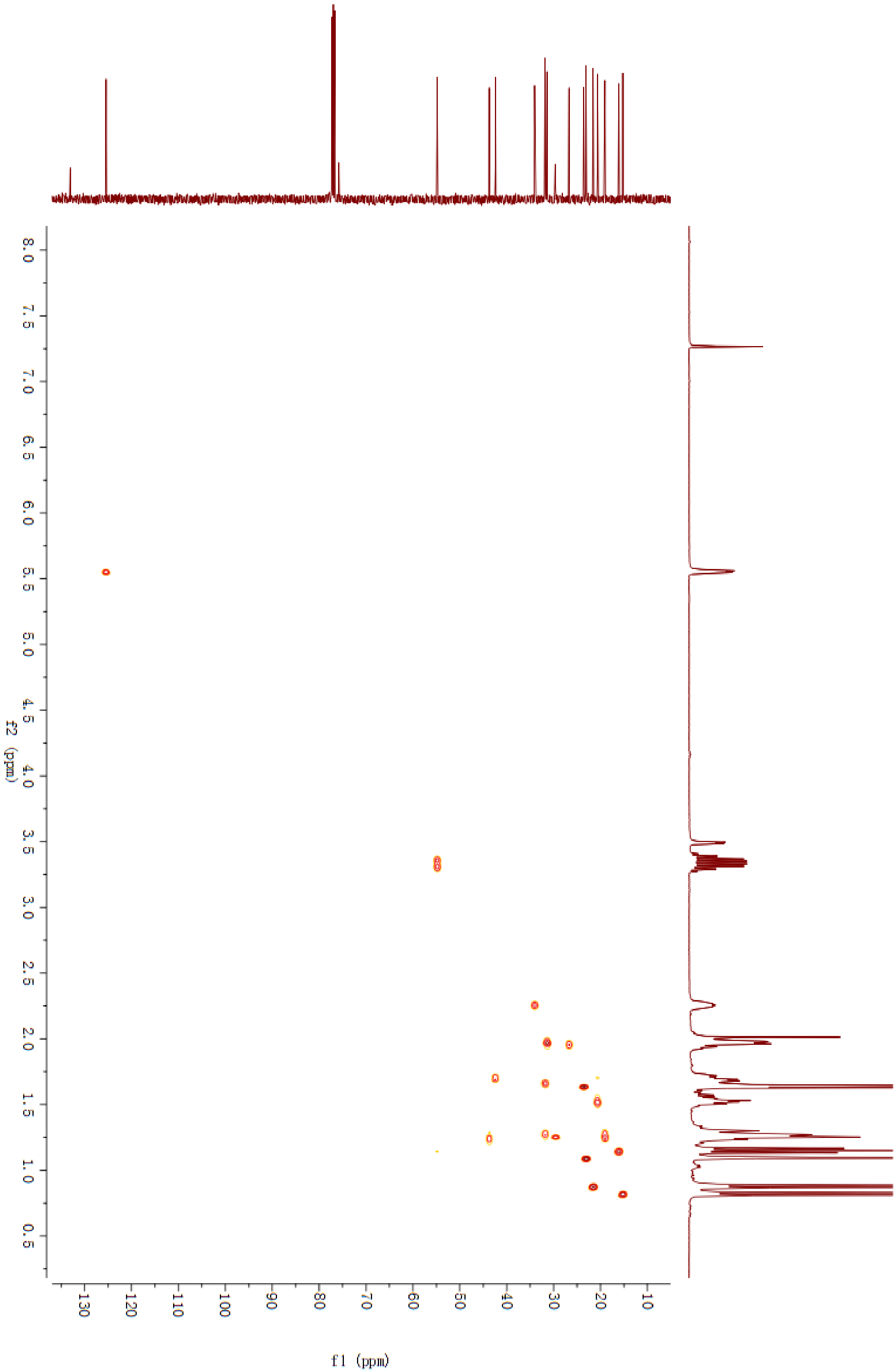

Figure 25S. HMBC of compound **4**.

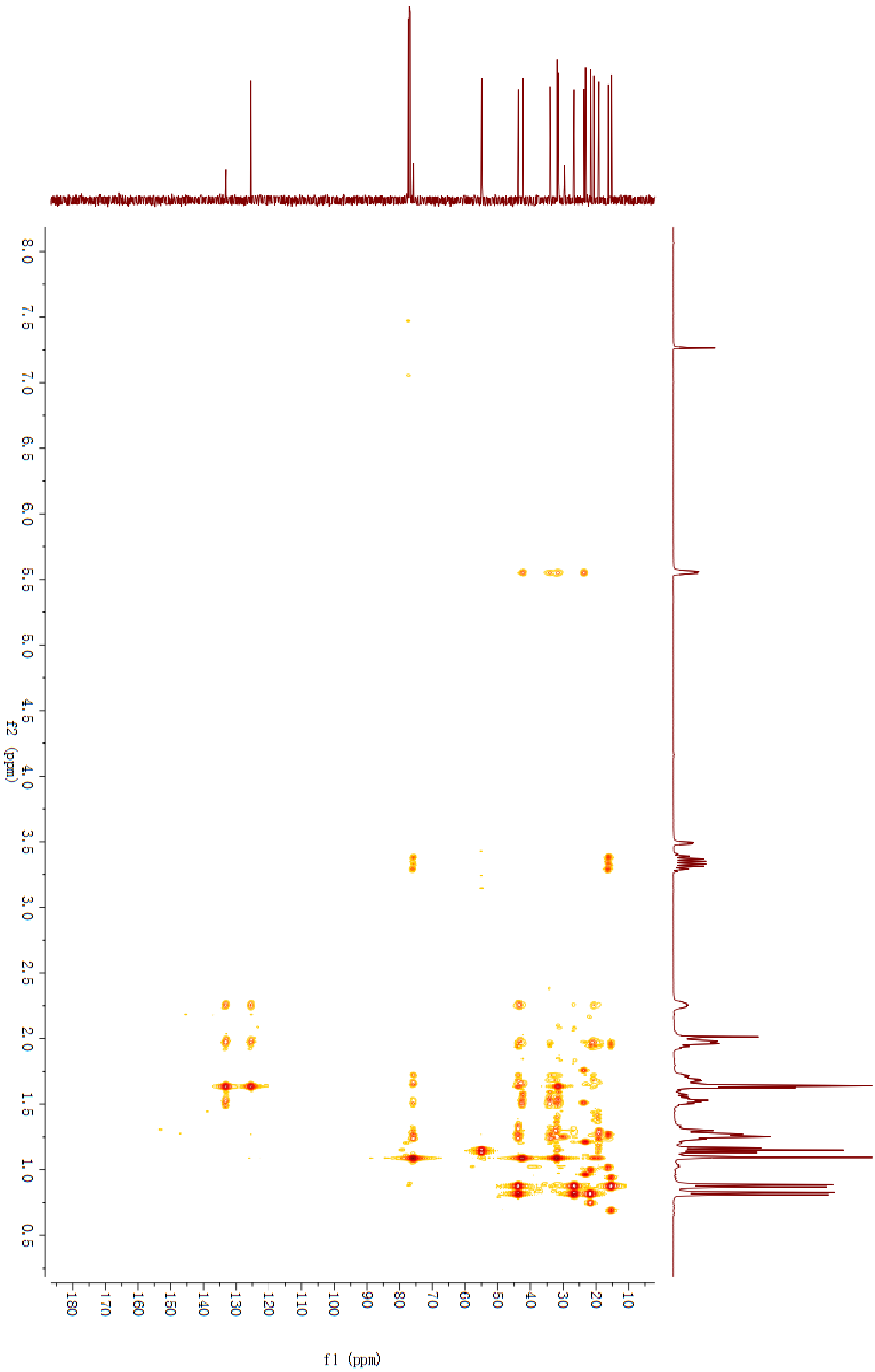

Figure 26S. H-H COSY of compound **4**.

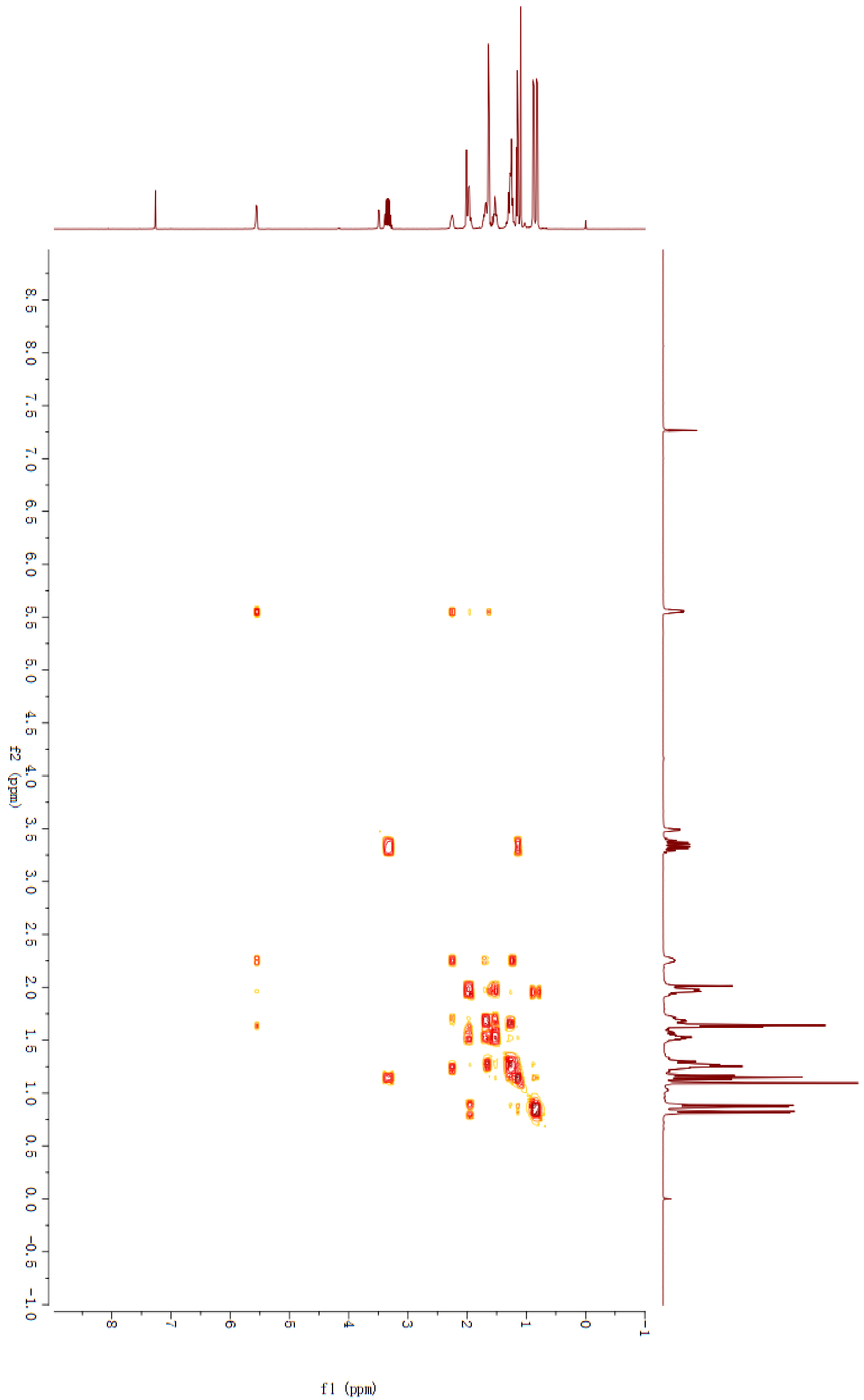


Figure 27S. ROESY of compound **4**.

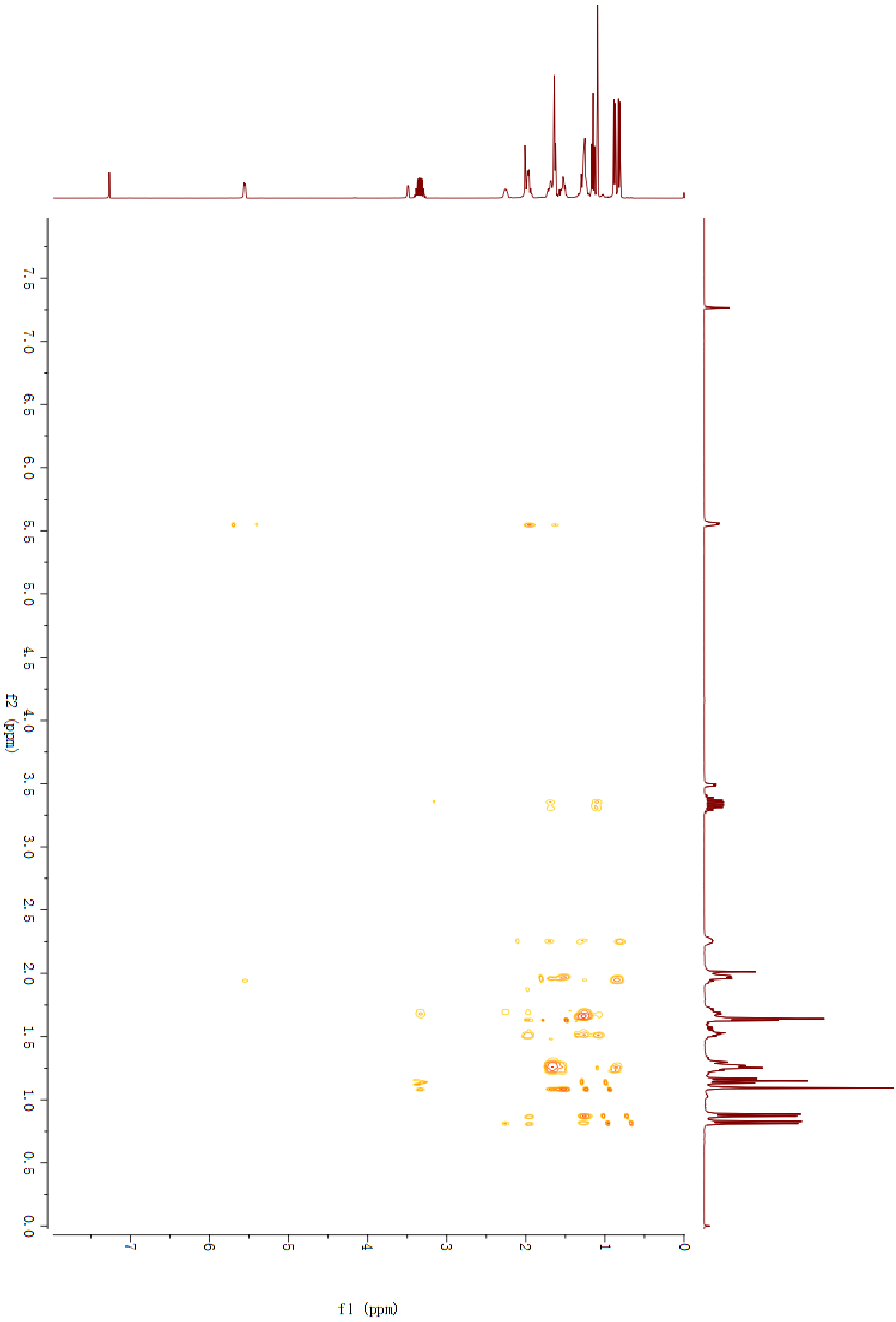
f2 (ppm)
f1 (ppm)

Figure 28S. HRESIMS of compound **4**.

Acq. Date: Thursday, November 24, 2011 Acq. Time: 16:00

Sample Name: 111124ESIA Ddcf3

## Elemental composition calculator

Target m/z: +273.2198 amu
Tolerance: +10.0000 ppm
Result type: Elemental
Max num of results: 1000
Min DBE: -10.0000 Max DBE: +60.0000
Electron state: OddAndEven
Num of charges: 0
Add water: N/A
Add proton: N/A
File Name: 111124ESIA Ddcf3.wiff

| | Elements | Min Number | Max Number |
|---|---|---|---|
| 1 | Br | 0 | 0 |
| 2 | C | 0 | 200 |
| 3 | Cl | 0 | 0 |
| 4 | F | 0 | 0 |
| 5 | H | 0 | 400 |
| 6 | I | 0 | 0 |
| 7 | K | 0 | 0 |
| 8 | N | 0 | 0 |
| 9 | Na | 1 | 1 |
| 10 | O | 0 | 1 |
| 11 | P | 0 | 0 |
| 12 | Pt | 0 | 0 |
| 13 | S | 0 | 0 |
| 14 | Si | 0 | 0 |

Acq. Date: Thursday, November 24, 2011 Acq. Time: 16:00

Sample Name: 111124ESIA Ddcf3

| | Formula | Calculated m/z (amu) | mDa Error | PPM Error | DBE |
|---|---|---|---|---|---|
| 1 | C17 H30 O Na | 273.2194 | 0.3644 | 1.3339 | 2.5 |
